# The impact of state- versus county-level mask mandates on economic activity during the COVID-19 pandemic

**DOI:** 10.1371/journal.pone.0332243

**Published:** 2026-09-03

**Authors:** Nathan Seegert, Maclean Gaulin, Nathorn Chaiyakunapruk, Francisco Navarro-Sanchez, Danielle Nguyen, Mu-Jeung Yang, Richard E. Nelson

**Affiliations:** 1 Department of Finance, Northeastern University, Massachusetts, United States of America; 2 School of Accounting, University of Utah, Utah, United States of America; 3 Department of Pharmacotherapy, University of Utah, Utah, United States of America; 4 Department of Economics, Allegheny College, Pennsylvania, United States of America; 5 Department of Economics, University of Oklahoma, Oklahoma, United States of America; 6 IDEAS Center, Veterans Affairs Salt Lake City Health Care System, Salt Lake City, United States of America; 7 Division of Epidemiology, University of Utah, Utah, United States of America; Gachon University, KOREA, REPUBLIC OF

## Abstract

**Background:**

During the COVID-19 pandemic, policy makers used mask mandates as a tool to signal that, due to the amount of virus circulating at the time, social behaviors might be risky. Because of the geographically specific nature of disease transmission information, these signals would be more informative in smaller geographic units. The objective of this study was to estimate the impact of policy information revelation by quantifying the impact of state versus county mask mandates on economic activity.

**Methods:**

We constructed a longitudinal dataset of US counties from April to September 2020 including the period immediately surrounding a COVID-19 mask mandate policy. In our primary analysis, we used a regression discontinuity approach with economic activity measured as county-level cell phone mobility and credit card spending.

**Results:**

We found that state mask mandates were associated with an increase in economic activity (coefficient = 1.045, 95% CI: 0.44,1.65) and spending per person (coefficient = $143, 95% CI: $102, $185). On the other hand, we found no statistically significant effects following county mask mandates.

**Conclusion:**

Using variation in the level of government enacting a policy, we find that mobility and spending are higher after a US state mask mandate than a county mandate, consistent with information revelation.

## Introduction

Throughout 2020, the federal U.S. government largely left public safety measures in response to the COVID-19 pandemic up to the states and counties. One such public safety measure was mandating the wearing of protective masks to prevent viral transmission through airborne particles and droplets. Riverside County, California, enacted the first county mask mandate in the U.S. at the beginning of April 2020. The first state mask mandate was put in place by New York state on April 17 and the last state mandate was put in place by Mississippi at the beginning of August 2020. From the beginning of April 2020 to the beginning of September 2020, 34 states plus Washington D.C. enacted state-wide mask-wearing mandates. Additionally, from April to September, 221 counties across 33 states put in place county-wide mask-wearing mandates.

Almost from the beginning, mask mandate policies were controversial and sparked a huge debate concerning their utility. However, the patchwork approach to mask mandate adoption provided heterogeneity in the timing and the level of government policy interventions. Such heterogeneity offers a natural laboratory to study the public’s reactions to public safety regulations.

In the years since the beginning of the COVID-19 pandemic, a number of studies have utilized this natural experiment to examine the impact of mask mandates on health outcomes. There is a growing body of evidence documenting the protective effect that these policies had on cases, hospitalizations, and deaths [1–4]. However, to our knowledge, no studies have focused on the association between mask mandates and economic activity. As motivation for examining this relationship, it is helpful to consider the mechanism by which mask mandates might influence economic activity, which we measure as cell phone mobility and credit card spending. While the primary purpose of these policies was to impact disease transmission, they also serve as a mechanism of signaling information from policy makers to the general public.

It is often the case that there is an information asymmetry between two parties [5]. In other words, one party knows more than the other. The more knowledgeable party can, however, reduce this asymmetry through actions that serve as signals for the other party. In economics, this is called signaling theory. Early in the pandemic, the vast majority of the US general public had not lived through a public health emergency on the scale of a global pandemic and, therefore, there was a great deal of uncertainty as to how safe it was to engage in typical consumer behavior such as going to a crowded shopping mall, grocery store, or restaurant. Mask mandates were a tool that policy makers could use to convey information to their constituents in two potential ways, each of which may have a different impact on economic activity. First, mask mandates increase safety from infection by increasing the use of a transmission mitigation tool (i.e., a mask) by the general public. Through this direct effect, mask mandates could lead to an increase in economic activity because individuals in the community perceive a lower risk of infection. Second, mask mandates can be a signal that, due to the amount of COVID-19 circulating at the time, social behaviors might be risky. In this case, mask mandates may lead to a decrease in economic activity. In addition, because of the geographically specific nature of disease transmission information, the information conveyed by these mask mandates may have a different effect if they are enacted in smaller vs. larger geographic units.

The theoretical framework of this paper draws on the economics of signaling and information disclosure. Akerlof [6] and Spence [7] establish the foundational insight that observable actions by informed parties transmit private information to less-informed parties and alter their behavior accordingly. Grossman and Stiglitz [8] extend this logic to show that agents rationally expend resources to act on information precisely because policies and prices are imperfect aggregators of what the informed party knows. In our setting, government mask mandates function as credible signals: policymakers, who have access to localized disease surveillance data, reveal their private information about transmission risk through the act of mandate adoption. Cho and Kreps [9] provide the equilibrium foundation for this interpretation—agents correctly infer that a mandate would only be enacted when conditions warranted it, since a policymaker with no cause for concern would not bear the political cost of a mandate. A key insight from Morris and Shin [10] is then directly applicable: the precision of a public signal determines how strongly agents update their beliefs and coordinate behavior. A county-level mandate is a more precise signal than a state-level mandate because it draws on geographically finer information, leading agents to update beliefs about local transmission risk more sharply. Milgrom [11] reinforces this logic through the unraveling result: when policymakers have private information and disclosure is credible, constituents infer the worst from inaction, making the decision to mandate highly informative. This precision, however, cuts both ways—a stronger signal of risk leads agents to curtail economic activity, partially offsetting the direct safety benefit of the mandate. The empirical literature on mandatory disclosure supports this behavioral response: Jin and Leslie [12] show that county-mandated restaurant hygiene grade cards caused consumers to reallocate spending in response to revealed health risk, and Dranove and Jin [13] document more broadly that government-mandated disclosure of health and quality information generates substantial and often unintended behavioral consequences. The divergence we document between state and county mandates is a direct implication of signal precision—more geographically targeted policies are more informative, produce larger belief updating, and in the case of mask mandates, generate a risk-avoidance response strong enough to reduce mobility and spending relative to the less precise state-level signal.

The objective of this national, retrospective cohort study was to estimate the impact of policy information revelation by quantifying the impact of all state and county mask mandates from April 2020 to September 2020 on economic activity. In particular, we empirically tested whether the signal role of mask mandates was stronger for different levels of government enactment. In contrast, the direct effect should have a simar effect across levels of government enactment if policy uptake is similar. Our hypothesis was that information revelation is higher when a county (a lower level of government) enacts a policy than when a state does. The insight here is that individuals learn more about infection risk when a county enacts the mask mandate than when the state does so because the information used by the county government is more geographically local.

## Methods

The analysis is built on a county-day panel dataset constructed from multiple administrative and commercial sources, covering the 25-day window before and 25-day window after each county’s relevant mask mandate and spanning April to September 2020. The sample encompasses all US counties subject to a state-level mandate—34 states plus Washington D.C., comprising the large majority of the US population—as well as 221 counties across 33 states that enacted county-level mandates independently of or prior to statewide action. Constructing the dataset at the county-day level allows the analysis to exploit fine-grained temporal and geographic variation in mandate timing, which is central to each of the three identification strategies. The patchwork nature of US pandemic policy—with states and counties acting independently at varying times—provides the quasi-experimental variation that underpins the empirical design. Card and Krueger [14] established the template for exploiting such policy heterogeneity across jurisdictions, and subsequent work by Bertrand, Duflo, and Mullainathan [15] demonstrated the importance of clustering standard errors to account for serial correlation in panel policy studies. This longitudinal structure, covering tens of thousands of county-day observations per subsample, provides sufficient statistical power to detect economically meaningful effects while absorbing the rich set of confounders described below.

### Data sources

We combined high-frequency (daily) data on economic activity, COVID-19 case growth, mask mandates, and other policies at the county level. Economic activity is measured using cellphone- GPS-based mobility data from Google and credit card transaction data from Safegraph/Facteus. Google provides this mobility data for different geographic locations and different categories of points of interest (https://www.google.com/covid19/mobility/). The Facteus data contains credit card spending data from multiple payment processing companies but only covers a subset of processed spending. For example, these data provide information on spending based on residence and do not distinguish between online versus in-person spending. We construct the dataset at the county level because that is the finest level we can obtain for many sources.

We use data from *The New York Times* on COVID-19 case growth at the county level. To measure mask mandates, we manually collected county-level data on mask mandates issued either by the state or the county. We also manually collected other COVID-19 policies such as limits on gatherings or school, restaurants, and bar closures, by going to each counties website, which we combined with similar data from Killeen et al. [16].

### Independent variables

The key independent variable in our regression discontinuity analyses was an indicator for post-mask mandate. We also controlled for important covariates that serve as confounders in the relationship between mask mandates and economic activity. These covariates included indicators for whether the majority of votes cast in the 2016 presidential election were for the Democratic Party rather than the Republican Party. Political affiliation is an important confounder in this setting for several reasons. First, Allcott et al. [17] document large and persistent partisan gaps in voluntary social distancing behavior during the COVID-19 pandemic, with residents of Democratic-leaning counties substantially more likely to reduce mobility and adopt protective behaviors independent of any formal mandate—meaning that baseline economic activity may differ systematically by political composition before a mandate ever takes effect. Second, because voluntary behavioral responses of this kind can reduce the measured discontinuity at the mandate threshold, failing to control for political affiliation would attenuate estimates in Democratic counties and inflate them in Republican counties, introducing differential bias across the two groups we compare. Third, political leaning correlates directly with the level of government enacting the mandate: in our sample, 45% of county-mandate counties voted Democratic compared to only 18% of state-mandate counties, meaning that partisan composition is a confounder not only of the mandate–activity relationship but specifically of the state-versus-county comparison that is central to our identification strategy.

The covariates also included indicators for whether the number of COVID-19 cases on the day of the mask mandate was above the median value of cases for the day that counties implemented mask mandates, more than 50% of the county population lives in an urban area, and more than 70% of survey respondents indicated that they wore a mask frequently at the time of being surveyed. In our difference-in-difference analyses, we controlled for a) whether retail, restaurants, bars, and schools are subject to restrictions, b) whether gatherings are limited to 10 or more people, 50 or more people, or thresholds of more than 50 people, and c) whether a stay-at-home order is in effect. We used two approaches to control for temporal trends. First, we use a third-order local polynomial to estimate the temporal trends. Second, we use event-time fixed effects to provide a flexible time trend before and after the mandate is enacted.

### Outcome variables

Economic activity was measured as county-level cell phone mobility and credit card spending. We focus on economically relevant categories, such as mobility for work, grocery shopping, retail shopping (including restaurants), and transportation (such as public transit). We exclude categories such as “parks,” since outdoor disease transmission is less common and mobility within parks has increased in some states during COVID-19. The Google mobility measures provide a daily- frequency comparison of mobility relative to the same calendar day in 2019, to control for general seasonal patterns. To put the mobility data in context, a 10-percentage point increase in mobility is associated with a two percentage point reduction in state unemployment rates [18]. From the Facteus credit card data we calculated a “spending per person per month” variable by scaling up the transactions reported in the dataset.

### Statistical analyses

We employ multiple empirical strategies, including a regression discontinuity (RD) design and a difference-in-differences (DiD) approach. The RD design exploits the discrete timing of mask mandate implementation by comparing activity in counties immediately before and after the policy change, under the assumption that unobserved determinants of activity evolve smoothly at the cutoff. This allows us to isolate the local effect of mandate adoption. We then contrast these discontinuous changes between counties subject to county-level mandates and those subject to state-level mandates.

The DiD approach complements the RD analysis by comparing trends in activity between counties with state and county mask mandates over a longer horizon. Prior to mandate adoption, counties that ultimately receive state mandates exhibit similar activity trends to those with county mandates, supporting the parallel trends assumption. The DiD estimates capture differential changes in activity following mandate implementation, while staggered adoption across counties provides additional identifying variation and mitigates concerns about confounding time-varying shocks. Together, these methods allow us to assess whether county and state mandates differ in their signaling effects on economic activity.

#### Regression discontinuity.

The primary empirical strategy is a regression discontinuity (RD) design that exploits the discrete timing of mask mandate adoption. The core identifying assumption is that unobserved determinants of economic activity evolve smoothly through the mandate date—so that any discontinuous jump in the outcome at that threshold is attributable to the policy itself. This assumption is plausible here because mandates were discrete legal acts whose exact dates reflected administrative and political processes rather than sharp breaks in underlying economic conditions, and the paper verifies the absence of significant pre-trend discontinuities through formal tests. The RD estimating equation for county *c* at event-time *t* (measured in days relative to mandate date t*_c_) is:


$$\begin{array}{c} Y_{ct}\;=\;\alpha \;+\;\beta \cdot {Post}_{ct}\;+\;f(t\;-\;t_{c}^{*})\;+\;{X^{\prime }}_{ct}\gamma \;+\;\varepsilon_{ct} \end{array}$$

where *Y*_*ct*_ is economic activity or spending in county *c* on day *t*; Post_*ct*_ is an indicator equal to one on and after the mandate date; *f*(·) is a flexible function of days relative to the cutoff estimated as either a third-order local polynomial or event-time fixed effects; X_*ct*_ is the vector of county-level covariates; and ε_*ct*_ is the error term. The coefficient β captures the local average treatment effect at the mandate threshold. Following Imbens and Kalyanaraman [19], the bandwidth is selected optimally to minimize asymptotic mean squared error, balancing the variance cost of using fewer observations against the bias cost of including observations far from the threshold. Standard errors are clustered at the county level, and confidence intervals are bias-corrected and robust following Calonico, Cattaneo, and Titiunik [20], whose procedure corrects for the estimation error in the bias term that standard asymptotic theory ignores. The RD is estimated separately for the state-mandate subsample and the county-mandate subsample; the difference in point estimates, β̂^*state*^ − β̂^*county*^, is the central quantity of interest and is reported with its own standard error in Table 2.

The RD design is well-suited to this setting for reasons extensively documented in the methodological literature. Lee and Lemieux [21] demonstrate that under the smoothness assumption, the RD estimator identifies a causal treatment effect without requiring random assignment of treatment status—it achieves internal validity comparable to a randomized experiment in the neighborhood of the threshold. This is particularly valuable here because counties that enacted mandates are not a random draw from the population: they tend to be more urban, more politically Democratic, and to have experienced higher COVID-19 case burdens at the time of adoption (Table 1). By focusing only on the within-county change around the mandate date, the RD controls nonparametrically for all permanent differences between mandate and non-mandate counties. Imbens and Lemieux [22] provide a practitioner’s guide to RD implementation and emphasize the importance of checking for discontinuities in predetermined covariates at the threshold; if covariates also jump at the mandate date, the smoothness assumption is violated. The paper’s finding of stable covariate profiles in the neighborhood of the mandate date supports the design’s identifying assumption. Hahn, Todd, and Van der Klaauw [23] establish the formal nonparametric identification conditions for RD, showing that the estimator is consistent under local continuity of potential outcomes, which is substantively reasonable given the day-to-day smoothness of economic activity absent a discrete policy shock. The nonparametric implementation used here, following Imbens and Kalyanaraman [19] and Calonico, Cattaneo, and Titiunik [20], avoids the sensitivity of parametric polynomial approaches to the order of the polynomial and the choice of functional form—a concern documented by Gelman and Imbens [24] in their critique of high-order polynomial RD specifications.

**Table 1:** Summary statistics.

Type of mandate								
	Counties with a state mask mandate				Counties with a county mask mandate			
	Mean	Median	Std Dev.	*N*	Mean	Median	Std Dev.	*N*
**Activity**	-14.18	-12.25	16.77	39,828	-16.19	-13.67	17.66	4,975
**Spend/month**	1159.98	751.20	1409.25	37,994	1128.70	846.37	1073.68	4,671
**Cases/pop**	12.05	4.25	26.03	44,270	25.07	16.47	30.81	5,085
**Comply county**	0.04	0.00	0.19	44,578	0.03	0.00	0.16	5,232
**Democrat county**	0.18	0.00	0.38	44,578	0.45	0.00	0.50	5,232
**High county**	0.53	1.00	0.50	44,578	0.77	1.00	0.42	5,232
**Urban county**	0.23	0.00	0.42	44,578	0.62	1.00	0.48	5,232

NOTE.— This table presents summary statistics for all variables used in the analysis. Each observation is a county- day for the 25 days prior to the mask mandate. *Activity* is relative to activity in 2019 in percent, using Google’s cell phone data. *Spend/month* is the average county-level credit card spending per person per month, scaled to the monthly level assuming one daily transaction per person on average. *Cases/capita* is the number of incident COVID-19 cases per day per 100,000 based on county-level population from the Census Bureau. *Comply county* is an indicator equal to one if at 70% of people surveyed stated that they wore a mask always or frequently, and zero otherwise. *Democrat county* is an indicator equal to one if the Democratic Party got more votes than the Republican Party in the 2016 presidential election, and zero otherwise. *High county* is an indicator equal to one if the number of cases on event-day 0 are above the median value of event-day 0 values, and zero otherwise. *Urban county* is an indicator equal to one if urban areas include more than 50% of the county population, and zero otherwise.

#### Difference-in-Difference.

**Fig 1:**
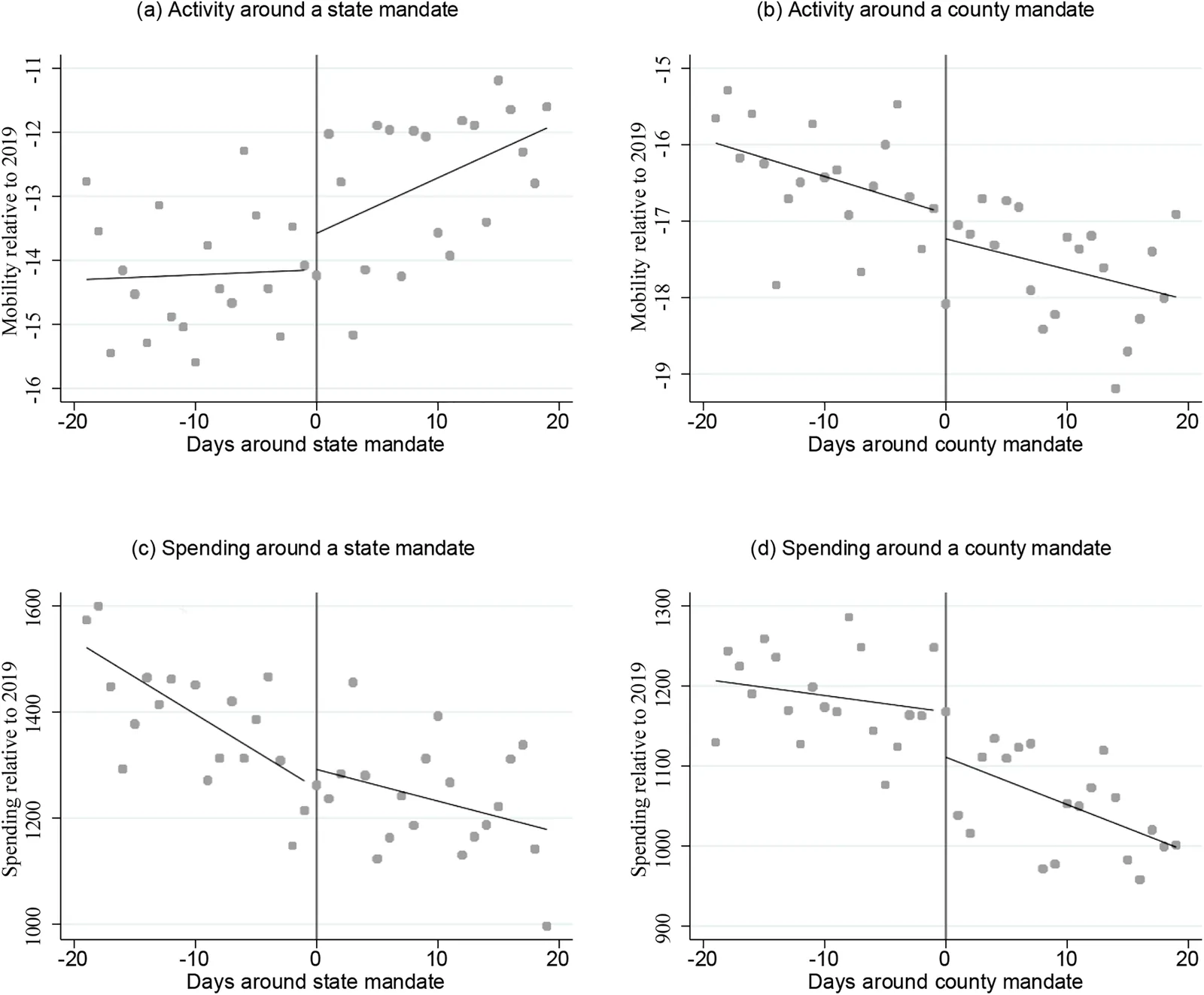
Activity and spending around a state and county mandate. Panels a and b report binned data and linear trends before and after a state mandate (panel a) and county mandate (panel b). Panels c and d report binned data and linear trends before and after a state mandate (panel c) and county mandate (panel d) mandate.

The difference-in-differences (DiD) design complements the RD by comparing activity in state-mandate counties against county-mandate counties over a longer horizon, capturing the cumulative informational difference between the two mandate types rather than only the local discontinuity at adoption. The estimating equation is:


$$\begin{array}{c} Y_{ct}\;=\;\alpha \;+\;\beta_{\text{1}}\cdot {State}_{c}\;+\;\beta_{\text{2}}\cdot {Post}_{ct}\;+\;\beta_{\text{3}}\cdot ({State}_{c}\;\times \;{Post}_{ct})\;+\;{X^{\prime }}_{ct}\gamma \;+\;\delta_{t}\;+\;\varepsilon_{ct} \end{array}$$

where State_*c*_ identifies counties subject to a state mandate, Post_*ct*_ is the post-mandate indicator, δ_*t*_ denotes event-time fixed effects, and X_*ct*_ includes the full set of time-varying policy controls. The coefficient of interest is β_*3*_, which captures the differential change in economic activity following a state mandate relative to a county mandate. The identifying assumption is parallel pre-trends: counties ultimately receiving state mandates must exhibit similar activity trajectories to those receiving county mandates in the period before mandate adoption. Fig 1 provides visual support for this assumption, showing broadly flat and similar pre-mandate trends across both groups; formal pre-trend tests further corroborate this. Heterogeneous treatment effects are modeled following Wooldridge [25] by interacting both State_*c*_ and Post_*ct*_ with county-type indicators for compliance rates, political leaning, prior COVID-19 case burden, and urban density, ensuring that the DiD estimate does not mask important cross-sectional variation in behavioral responses to mandates.

Following Wooldridge [25], we explicitly model these heterogeneous treatment effects by including interactions of *state mandate* and *mask mandate* with different types of counties. We consider counties that are more likely to comply with mask mandates, vote Democratic, had higher rates of COVID-19 before the mandate, and are urban.

#### Synthetic control.

The synthetic control method, introduced by Abadie and Gardeazabal [26] and formalized by Abadie, Diamond, and Hainmueller (2010) [27], constructs a data-driven counterfactual for each treated unit by finding a convex combination of untreated units whose pre-treatment outcome trajectory most closely matches the treated unit. Formally, the synthetic control weights *w*_*j*_ ≥ 0 with Σ_*j*_ *w*_*j*_ = 1 are chosen to minimize the pre-mandate discrepancy:


$$\begin{array}{c} {{\text{min}}_{\left\{wj\right\}}\;\Sigma_{\tau <\text{0}}\;(Y_{c\tau }\;-\;\Sigma_{j}\;w_{j}\;Y_{j\tau })}^{\text{2}}\text{ subject to }w_{j}\;\geq \;\text{0},\;\Sigma_{j}\;w_{j}\;=\;\text{1} \end{array}$$

Weights are selected by matching on pre-period means of county population, economic activity, COVID-19 case growth, and credit card spending. The post-mandate treatment effect for county *c* at time *t* is then τ̂_*ct*_ = *Y*_*ct*_ − Σ_*j*_ *w*_*j*_ *Y*_*jt*_. The aggregate effect at each horizon is the average of unit-level estimates across treated counties. This approach is applied separately at 25-day and 75-day post-mandate horizons to assess both the immediate and medium-run persistence of effects. The restriction *w*_*j*_ ≥ 0 prevents extrapolation beyond the support of the control group, a desirable property when treated and control units differ substantially on observables, as is the case here.

Abadie [28] provides a comprehensive review of synthetic control methods and their advantages relative to difference-in-differences. The key benefit is that the synthetic control does not require the parallel trends assumption to hold globally across all treated and control units; instead, it constructs a tailored counterfactual for each treated unit, matching its specific pre-mandate outcome trajectory rather than assuming all untreated counties share the same baseline trend. This is particularly important here because counties subject to county-level mandates differ systematically from those covered by state mandates along multiple observable dimensions (Table 1): they are more urban, more politically Democratic, and faced COVID-19 case rates more than twice as high. The synthetic control absorbs these differences by construction. Abadie, Diamond, and Hainmueller [29] extend the framework to settings with multiple treated units and discuss inference based on permutation distributions, which are used here to assess statistical significance without relying on large-sample approximations. A further advantage noted by Abadie [28] is transparency: the weights make the counterfactual construction explicit and verifiable, and the quality of pre-treatment fit provides a direct, visible diagnostic for the method’s credibility. A poor pre-treatment fit would signal that no adequate control group exists for a given treated county, alerting the researcher before any post-treatment comparison is made—a diagnostic not available in standard DiD.

#### Falsification tests.

Finally, we conducted a series of ’falsification tests’ in which we conducted analyses similar to our primary analyses but in subsets of data which we would not expect to find a relationship of interest.

The first falsification test was based on the timing of the mask mandates. In this analysis, we used the 2,188 counties with a county or state mandate and assigned their mandate to occur randomly between 25 and 100 days after the relevant mandate. If our main effects are explained by differences across counties and not the information revelation of the mask mandate policies, then we should find a similarly large, positive, and statistically significant treatment effect in this falsification test.

The second falsification test focused on the specific locations of the mask mandates. For this test, we selected all counties without a county or state mandate adjacent to a county that enacted one (or is in a state that enacted one). This resulted in 370 falsification counties. We assigned the same mask mandate enactment date for these falsification counties as that of the adjacent county. In cases in which there are multiple adjacent counties with mask mandates, we assigned the first date. We expect some spillover effects from the mask mandate to adjacent counties, but these effects likely bias us toward finding an effect due to these spillovers. For example, people in adjacent counties may shop in the nearby county with a mask mandate due to increased safety.

#### Alternative explorations and explanations.

We also provide an additional test of the policy information revelation mechanism using variation in a county’s population relative to the population in its state. The information revelation from a state mandate is weaker in counties that are small in population relative to their state because the disease transmission risks in the smaller county may be less correlated with the risks in the rest of the state. If this information revelation is an important mechanism, then economic activity after a state mandate should increase more for counties with small populations relative to their states. We tested this by interacting the state mandate and mask mandate indicator variables with the amount of population in a county’s state, not in the county. We called this variable the *net population.*

We then tested for alternative mechanisms by modeling treatment effect heterogeneity. These models control for “selection on gains,” such as Democratic states being more likely to implement state mandates than county mandates because Democratic governors expect mask mandates to be more effective than Republican governors. Specifically, we are concerned that some counties have stronger treatment effects from mask mandates and that those effects correlate with whether a county was subject to a state or county mandate.

## Results

**Fig 2:**
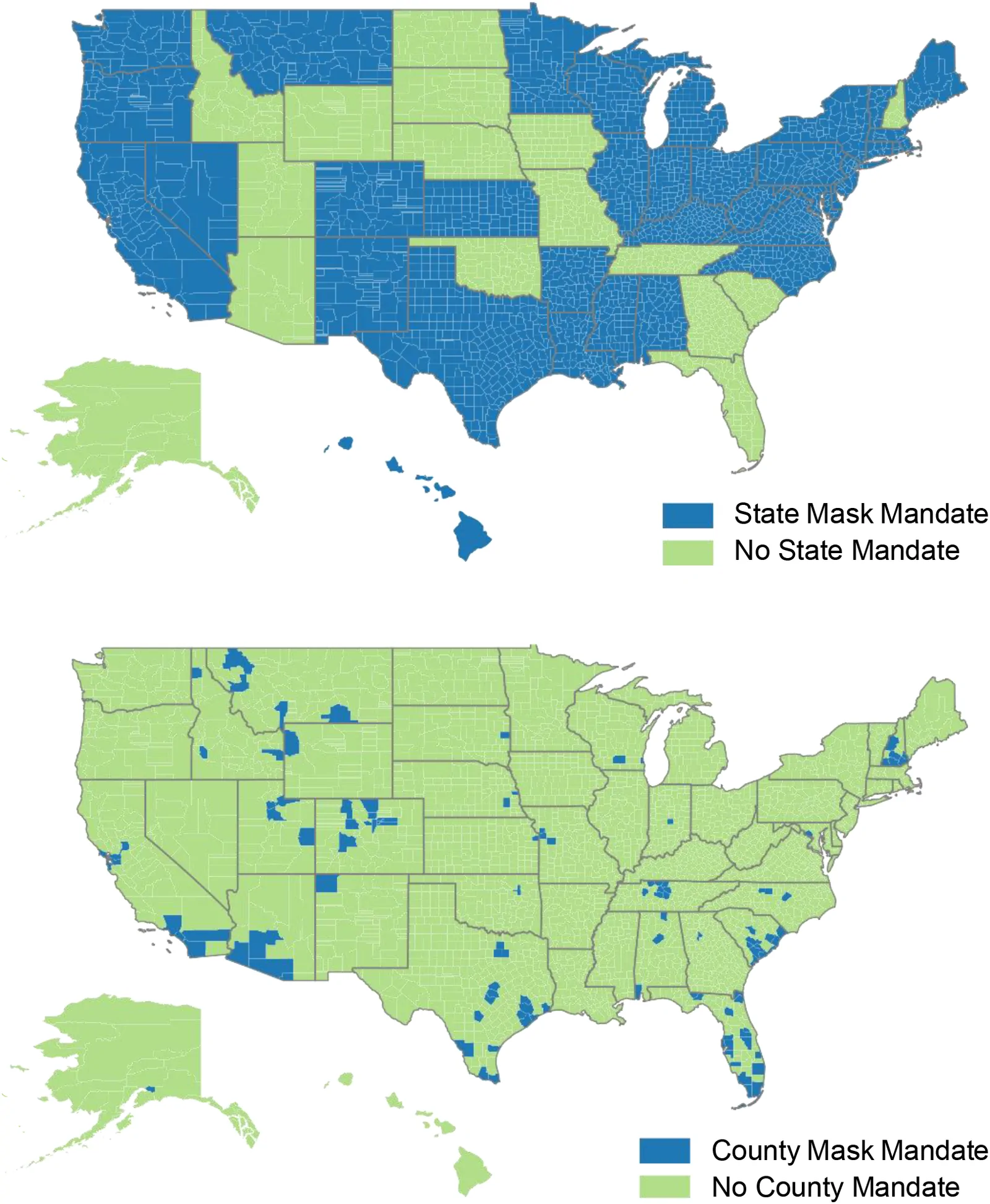
Mandatory Mask Mandates Map. Fig 1 shows maps of the county- and state-level mandates implemented at some point in our sample from April 2020 to September 2020. Many of the county-level mandates come from states that eventually also had a state-level mandate.

Fig 2 shows the geographic dispersion of state and county mandates during our study period. Summary statistics for key variables at the county level are reported in Table 1 stratified by whether the county had a state or a county mask mandate. Many more counties were subject to state-level mandates than county-level mandates. The average activity, measured as mobility at economically relevant activities, was 14% to 16% lower during our study period than for dates in 2019, reflecting the general reduction in economic activity during much of 2020 [18]. It is also noteworthy that COVID-19 case rates were more than twice as high in counties with county mask mandates (25.07%) compared to those with state mandates (12.05%). In addition, relative to counties subject to a county mandate, counties subject to a state mandate were more compliant with mask mandates (4% versus 3%), were less likely to vote for the Democratic candidate in the 2016 presidential election (18% versus 45%), and less urban (23% versus 62%).

We show how trends in our outcome variables change in the days surrounding a state or county mask mandate in Fig 2. Activity is measured by cell phone mobility data and spending is measured by credit card spending. Panel A shows that activity increased immediately after a state mandate with a flat average trend in the 20 days prior to the mandate followed by a sharp upward trend in the 20 days after the mandate. In contrast, Panel B shows that activity exhibited a similar negative average trend both in the 20 days prior to and the 20 days following a county mandate. We find a similar pattern in spending, with a slight increase after a state mandate and a decrease after a county mandate, albeit relative to a negative overall trend (Panels C and D). The mask mandates have two effects: they increase safety by increasing mask wearing in public, encouraging activity and spending, and they signal increased risk, discouraging activity and spending. On net, we find the signal is sufficiently weak with state mandates to lead to an increase in activity and spending, while the signal of risk is sufficiently strong with county mandates such that the information effect is larger than the effect of increased safety.

The results from regression discontinuity analyses estimating the impact of state and county mask mandates on overall activity and spending are shown in Table 2. State mandates were associated with an increase in activity of 1.045 (p *<* 0.01) and 0.345 (p *<* 0.01) when including a flexible or cubic time trend, respectively, but there was no statistically significant effect following a county mandate. Similarly, we find that spending per person per month increased by $143 (p *<* 0.01) and $44 (p *<* 0.01) after a state mandate, but no significant difference was seen following a county mandate.

**Table 2:** Regression discontinuity results of activity and spending by state and county mask mandates.

Regression Discontinuity (RD)								
Method:	Third-order local polynomial				Flexible time trend			
	Activity		Spend/month		Activity		Spend/month	
	State	County	State	County	State	County	State	County
	(1)	(2)	(3)	(4)	(5)	(6)	(7)	(8)
RD estimate	1.05***	-1.01	143.46***	-44.00	0.35***	-0.25	43.58***	5.24
	[0.44, 1.65]	[-2.70, 0.67]	[102.20, 184.70]	[-136.10, 48.11]	[0.13, 0.56]	[-0.76, 0.25]	[21.75, 65.42]	[-19.61, 30.10]
Observations	46,389	8,249	165,884	19,827	45,482	7,727	152,905	18,225
*RD_state_ −RD_county_*					0.60**		38.34**	
					[0.05, 1.15]		[5.43, 71.25]	

NOTE.— Table 2 reports regression discontinuity estimates days around a state (columns 1, 3, 5, 7) or county (columns 2, 4, 6, 8), mask mandates using flexible time trends (columns 5–8), and a cubic time trend (columns 1–4) for activity (columns 1, 2, 5, and 6) and spending (columns 3, 4, 7, and 8). *Activity* is relative to activity in 2019 in percent, using Google’s cell phone data. Standard errors clustered at the county level and 95% confidence intervals are presented in parentheses. We denote statistical significance by ^*^ *p <* 0*.*10, ^**^ *p <* 0*.*05, ^***^ *p* *<* 0*.*01.

The estimated RD coefficient of 1.05 for activity implies that, following a state mask mandate, county-level mobility increased by approximately 1.05 percentage points relative to the same calendar day in 2019. Given that average activity during our sample period was roughly 14–16 percent below 2019 levels, this effect corresponds to a non-trivial rebound in economic activity. In contrast, the estimated coefficient of −1.01 following county mask mandates indicates a small decline in activity of approximately one percentage point, though this estimate is not statistically distinguishable from zero. This suggests that county mandates did not generate meaningful changes in observed mobility around the time of implementation. For spending outcomes, the estimated coefficient of 143.46 implies an increase in average credit card spending of approximately $143 per person per month following a state mask mandate. Relative to the mean monthly spending of approximately $1,160 in counties subject to state mandates, this represents an increase of roughly 12 percent. No comparable increase is observed following county mandates.

**Fig 3:**
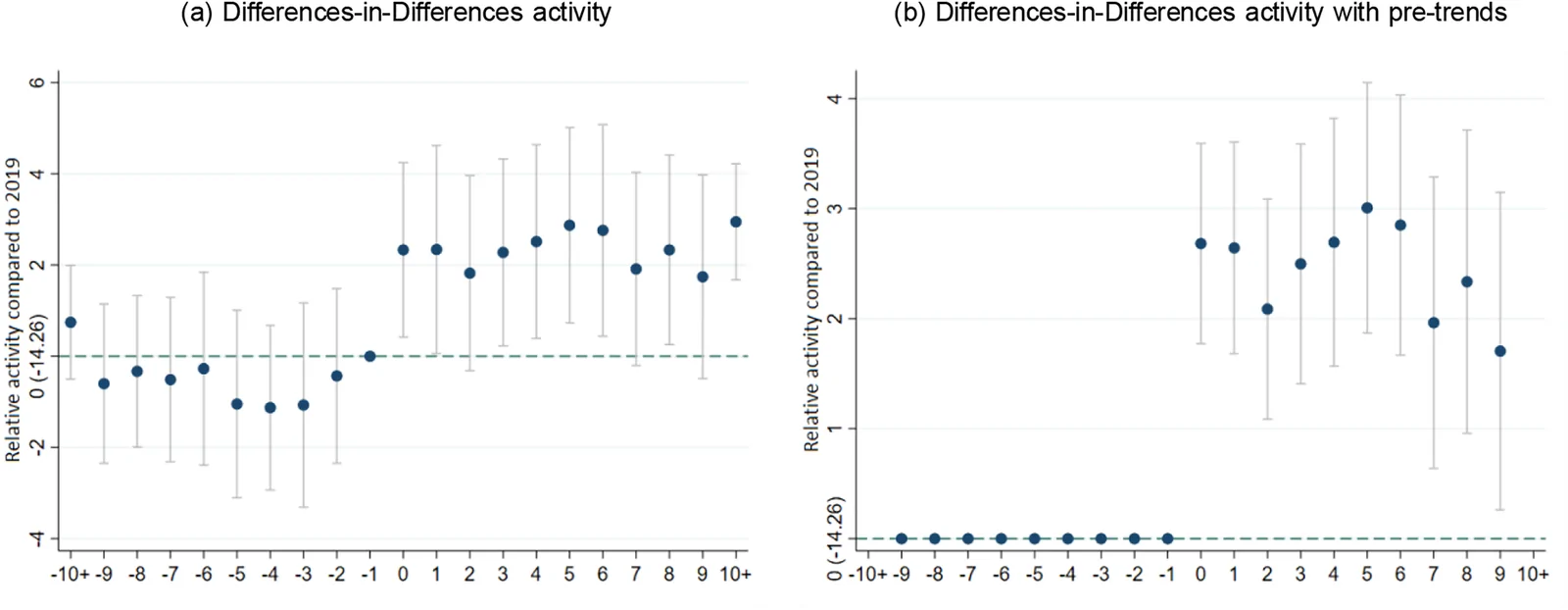
Activity and spending around a state and county mandate differences in differences. Panels a and b show *activity* after a state mask mandate, relative to a county mask mandate, using a panel event study design following Freyaldenhoven et al. [30]. The vertical axis displays the relative activity compared to 2019 after a state mandate, compared to a county mandate. Panel a uses county and date fixed effects. Panel b allows for a pre-trend as in Dobkin et al. [31].

We find the contrast across these panels is robust to a series of different specifications and identifying assumptions. This occurs in the absence of any significant difference in pre-treatment trends and even with the effects after mask adoption being persistent. In Fig 3, we present estimates with a flexible estimate of the pre-treatment trend subtracted from the outcome variable. We follow Freyaldenhoven et al. [30], who demonstrate that this method is robust to the possibility that there may be unobserved common trends too subtle to be reliably ruled out at standard levels of statistical significance.

In Table 3, we report results from the alternative modeling approaches. Using a difference-in- differences approach, we found that the increase in economic activity associated with state mandates compared to county mandates ranged from 3.2 (p *<* 0.01) to 2.9 (p *<* 0.01) percentage points. In the synthetic control approach, we found that economic activity was 0.79 (p *<* 0.05) and 1.57 (p *<* 0.01) percentage points higher over a 25- and 75-day time horizon, respectively. We found similar results for the spending outcome.

**Table 3:** Results from alternative regression specifications with activity and spending.

Panel A Dependent variable: Activity								
	Dif-in-Dif		Mechanism		Synth. Control		Falsification	
	No controls	FE	FE + Controls	Net pop.	Short run	Medium run	Time	Location
	(1)	(2)	(3)	(4)	(5)	(6)	(7)	(8)
State-mandate *×* Post-mandate	3.19^***^	3.28^***^	2.95^***^	1.73^***^	0.79^**^	1.57^***^	0.54	0.51
	[2.21, 4.16]	[2.32, 4.25]	[2.17, 3.73]	[0.65, 2.82]	[0.15, 1.44]	[0.89, 2.25]	[-0.48, 1.55]	[-0.60, 1.61]
State-mandate *×* Post-mandate *×* Net pop.				0.13^**^ [0.03, 0.23]				
Controls			✓	✓			✓	✓
County fixed effects		✓	✓	✓			✓	✓
Event time fixed effects		✓	✓	✓			✓	✓
Adj. R-Square	0.010	0.871	0.873	0.001	0.003	0.873	0.656	0.833
Observations	93,191	93,181	93,181	93,181	82,523	161,355	90,189	16,510
Panel B Dependent variable: Spend/month	Dif-in-Dif		Mechanism		Synth.Control		Falsification	
	No controls	FE	FE + Controls	Net pop.	Short run	Medium run	Time	Location
	(1)	(2)	(3)	(4)	(5)	(6)	(7)	(8)
State-mandate *×* Post-mandate	35.45^**^	55.89^***^	57.61^***^	3.87	18.81^**^	43.63^***^	-9.32	8.79
	[3.36, 67.54]	[29.87, 81.90]	[30.01, 85.20]	[-29.83, 37.57]	[3.41, 34.20]	[23.57, 63.69]	[-37.96, 19.31]	[-36.77, 54.35]
State-mandate *×* Post-mandate *×* Net pop.				2.24^**^ [0.27, 4.21]				
Controls			✓	✓			✓	✓
County fixed effects		✓	✓	✓			✓	✓
Event time fixed effects		✓	✓	✓			✓	✓
Adj. R-Square	0.002	0.862	0.864	0.883	0.001	0.002	0.841	0.839
Observations	88,229	88,228	88,228	49,123	75,670	130,746	87,828	11,923

NOTE.— This table reports alternative identification strategies for the dependent variables *Activity* in Panel A and *Spend/month* in Panel B. These methods include differences-in-differences (columns 1–3), a triple difference specification using variation in net population (column 4), synthetic controls (columns 5 and 6), and two placebo tests with random time (column 7) and counties that did not have a mask mandate (column 8). *Net population* (net pop.) is defined as the amount of the state’s population not in one’s county. Controls are used in columns 3, 4, 7, and 8 and include indicator variables for whether retail, restaurants, and bars are restricted, as well as restrictions to stay at home, school closings, and restrictions on gatherings of 10 or more people, 50 or more people, or thresholds of more than 50 people. *Activity* is relative to activity in 2019 in percent, using Google’s cell phone data. *State mandate* is an indicator equal to one if an order mandated at the state level is in place, and zero otherwise. *Mask mandate* is an indicator equal to one if an mask order is in place, and zero otherwise. *Spend/month* is the average county-level credit card spending per person per month, scaled to the monthly level assuming one daily transaction per person on average. Standard errors clustered at the county level and 95% confidence intervals are presented in parentheses. We denote statistical significance by ^*^ *p* *<* 0*.*10, ^**^ *p* *<* 0*.*05, ^***^ *p* *<* 0*.*01.

**Fig 4:**
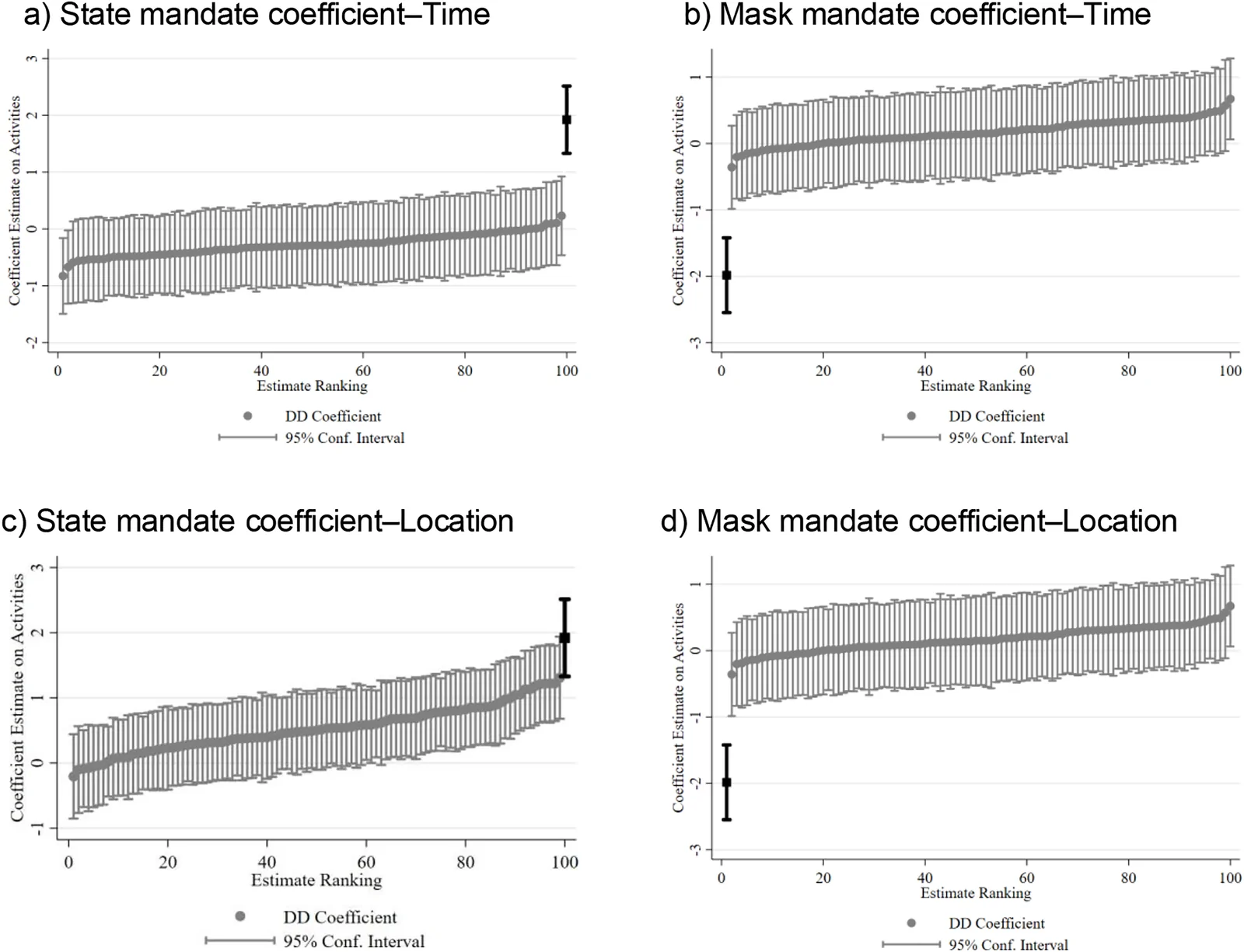
Time and location falsification tests. Fig 4 shows 100 coefficient estimates ordered by size: 99 from a placebo test (shown in gray) and 1 from our baseline estimate (shown in black). Each placebo estimate represents a different random draw of event times (Panels a and b) or locations (Panels c and d).

Panels A and B of Figure 4 depict coefficients from 99 falsification tests using different weeks on *state mandate* and *mask mandate*, respectively, sorted from smallest to largest in gray. Our baseline estimate appears in black. As can be seen, we find no evidence of an effect across these falsification tests. These tests suggest that our main findings are not driven by unobservable cross-sectional confounding factors or by chance. Column 7 of Table 3 reports the average coefficient across these 99 iterations from the time falsification test for the economic activity and spending outcomes, respectively.

Similarly, we found no large statistically significant effect in the location falsification tests, with coefficients reported in Panels C and D of Fig 4. Each falsification estimate represents a different random selection of adjacent counties. Our baseline result is the greatest effect with smaller confidence intervals, suggesting that this size is not due to chance or other confounding factors. A handful of the results in Panel C are positive and statistically significant which is consistent with some spillovers across counties. The relatively smaller size of these estimates suggests that other confounding factors are unlikely to lead to our estimate. Column 8 of Table 3 reports the average coefficient across these 99 iterations from the location falsification test for the economic activity and spending outcomes, respectively.

We find the interaction between state-mandate, post-mandate, and net population is positive and statistically significant for economic activity measured as mobility (Panel A) and credit card spending (Panel B) as shown in Table 3. These estimates suggest that for counties where the state mandate was less informative because they are a smaller share of the state’s population increased mobility and spending more. This evidence is consistent with the information revelation mechanism and inconsistent with other alternative explanations. Another advantage of this test is that it validates our main estimates while relying on different variation created by population differences. Specifically, it relies on variation across counties in states that all enacted state mandates. Therefore, this test helps to rule out alternative explanations about differences between counties that had mask mandates due to state or county mandates. Said differently, our results cannot be explained by unobserved differences in the counties that were subject to state and county mandates.

In Table 4, we show that our results are robust to allowing for heterogeneity in compliance, political leaning, prior beliefs based on COVID-19 case counts, and density. Specifically, these estimates suggest that our results cannot be explained by an alternative explanation holding that heterogeneity in treatment effects correlated with whether a county was subject to a state or county mask mandate.

**Table 4:** Alternative specifications of activity by state and county mask mandates.

	Baseline	Mask effectiveness		Beliefs	Density
Interaction:	None	Comply	Democrat	High Rate	Urban
	(1)	(2)	(3)	(4)	(5)
State mandate	1.921***	2.810***	2.400***	3.733***	1.945**
	(0.302)	(0.415)	(0.447)	(1.146)	(0.784)
Mask mandate	-1.985***	-1.949***	-1.398***	-1.593	-0.793
	(0.287)	(0.392)	(0.412)	(1.135)	(0.761)
State mandate *×* factor		0.134	0.661	-1.738	0.799
		(3.930)	(0.898)	(1.228)	(0.939)
Mask mandate *×* factor		1.279	-1.091	-0.373	-1.678*
		(3.796)	(0.812)	(1.200)	(0.889)
Controls	✓	✓	✓	✓	✓
County fixed effects	✓	✓	✓	✓	✓
Week fixed effects	✓	✓	✓	✓	✓
Adj. R-Square	0.873	0.831	0.831	0.832	0.831
Observations	100,938	100,938	100,938	100,938	100,938

NOTE.— *Activity* is relative to activity in 2019 in percent, using Google’s cell phone data. We consider a time period encompassing the 25 days before and after a mask mandate. *State mandate* is an indicator equal to one if an order mandated at the state level is in place, and zero otherwise. *Mask mandate* is an indicator equal to one if an mask order is in place, and zero otherwise. *Comply* is an indicator equal to one if 70% of people surveyed stated that they wear a mask always or frequently, and zero otherwise. *Democrat* is an indicator equal to one if the Democratic Party received more votes than the Republican Party in the 2016 Presidential Election, and zero otherwise. *High rate* is an indicator equal to one if the number of cases on event-day 0 is above the median value of event-day 0 values, and zero otherwise. *Urban* is an indicator equal to one if urban areas include more than 50% of the county population, and zero otherwise. Controls include indicator variables for whether retail, restaurants, and bars are restricted, as well as restrictions to stay at home, school closings, and restrictions on gatherings of 10 or more people, 50 or more people, or thresholds of more than 50 people. Standard errors clustered at the county level are in parentheses. We denote statistical significance by ^*^ *p* *<* 0*.*10, ^**^ *p* *<* 0*.*05, ^***^ *p* *<* 0*.*01

## Discussion and conclusion

In this paper, we quantify the information revelation of mask mandates during the COVID-19 pandemic by investigating differences in economic activity following county and state mandates. The effects of mask mandates were complicated; economic activity was unaffected by county mandate but increased following state mandates. We posit the difference in effects is due to differences in information revelation. Consistent with this information revelation mechanism, we find that activity, measured by cell phone mobility, is 1.9 percentage points greater after a state mandate than a county mandate. The key difference between these settings is that the information revelation from a county mandate is more local than a state mandate—implying that people should update their expectations more after a county rather than state mandate. Put differently, individuals learn more about the risks in their location when policies are enacted at a lower level of government. After a state-level mandate, information revelation will be smaller in a relatively small county compared to a relatively large county. Consistent with the information mechanism, we find that economic activity is smaller in counties with a higher share of a state’s population after a state mandate. For example, when Utah enacted its state mask mandate, the information revelation was larger in Salt Lake County, which accounts for 36% of the state’s population, than in Carbon County, which accounts for less than 1%.

In our context, we find that governments that considered the information revelation would be more likely to enact state rather than county mandates to increase safety and economic activity. We show that these unintended effects can be dampened or amplified depending on the level of government that enacts the policy, even when additional tests were done to help rule out other potential mechanisms. Together, the evidence suggests a policy-relevant role for information revelation.

Our paper contributes to a growing literature on unintended policy consequences by highlighting the importance of information revelation. Our results also contribute to the recent literature on the role of different crisis management policies on health outcomes and economic activity during the COVID-19 pandemic (see for example [17,18,32–39]). Our estimates compare and contrast the effectiveness of policies implemented at different levels of government, complementing the literature that has mostly focused on the effectiveness of a policy at a given level. We highlight that which level of government implements the policy has a large impact on the effectiveness of the policy.

Government policies result in a variety of intended and unintended effects. We have shown how information revealed by government policies can have countervailing effects to their intended consequences. In the context of mask mandates, the policy improves safety and increases economic activity, however, the information revelation increases people’s perceived risk and decreases their economic activity. When more information about COVID-19 risk was revealed, individuals increased their perceived risk and decreased their economic activity.

It is important to point out several limitations to our study. First, credit card spending and cell phone data – while useful measures of purchasing and mobility, respectively – do not capture the entirety of economic activity. In addition, the credit card spending does not distinguish between online and offline spending, which could bias our results in either direction. For example, if mask mandates increased the perceived risk of in-person interactions, some consumers may have substituted online for offline spending but with no net change in total spending. This could inflate our spending coefficient relative to the true effect. On the other hand, the substituted online spending could be done through vendors whose data is not captured in the Facteus data set. This could lead to a reduction in the magnitude of the effect estimate. We are somewhat reassured that this substitution effect is not a substantial driver of our results because both the spending and activity effects are in the same direction. Second, because our study relies on real-world, observational data, there is always a concern that our estimated effects are biased due to unmeasured confounding. While this may be true, we feel that our thorough approach minimizes this risk. For instance, we employed several different statistical approaches and conducted a number of falsification tests all of which gave strong support for our overall findings. Finally, we document an intriguing difference in effects when the policy is implemented at the state or county level. We conjecture that this difference is due to information revelation and provide supportive evidence for this explanation. However, there could be other explanations that we have not considered.

In conclusion, the enactment of a policy reveals information. In our context, when a state or a county enacted a mask mandate, it revealed information about the risks of COVID-19. The direct effect of enacting a mask mandate was to increase safety by decreasing transmission. The information revelation of enacting a mask mandate increased people’s expectations of transmission risk because they reasoned correctly that policymakers would only enact a mandate when risks were high.

These findings can be informative to policy makers as they underscore the importance of understanding the messages (which may be secondary or even unintended) that are conveyed to constituents through enacted policies. And, as was the case with mask mandates during the early days of the COVID-19 pandemic, this information can lead to behavior changes that can have substantial economic consequences.

## Supporting information

S1 File Analysis dataset. (CSV)
