# Supplementary material for: The impact of state- versus county-level mask mandates on economic activity during the COVID-19 pandemic
Source: PLoS One. 2026 Sep 3;21(9):e0332243. doi: 10.1371/journal.pone.0332243 (PMC13541194; doi:10.1371/journal.pone.0332243)
Supplement: Supplementary file 1 [file pone.0332243.s005.docx]

**Response to Editor and Reviewer Comments**

*Manuscript Title:* The Impact of State- versus County-Level Mask Mandates on Economic Activity During the COVID-19 Pandemic

*Journal: PLOS One*

*Manuscript ID:* PONE-D-25-46516R1

We sincerely thank the Editor and reviewers for their continued careful reading of our manuscript and for the constructive feedback provided in this round of review. We have revised the manuscript in response to all comments raised. Below, we address each point in turn. Our revisions are described in detail, and all changes to the manuscript are indicated in the revised version by track changes.

**Responses to Editor Comments**

**Editor Comment:** Response to Reviewer #2’s comment is not provided. Please address Reviewer #2’s concern.

**Response:**

Thank you for catching this and apologies for missing this on our previous submission. Please find our response below.

**Editor Comment:** I did not mean to split Discussion and Conclusion independently. Please revise the last section title as “Discussion and Conclusion”

**Response:**

We have made this change in the revised version of the manuscript.

**Responses to Reviewer #2 Comment**

**Comment:** The authors added a limitation regarding not being able to distinguish between online and offline spending. Briefly discuss how this might bias the spending per person - results. (ex. potential substitution bias).

**Response:** We appreciate the reviewer asking for more detail on this limitation. We have expanded the description of the consequences of our inability to distinguish between online and offline spending in the discussion section. To briefly summarize, the direction of the possible substitution bias could go either way. Mask mandates may increase the perceived risk of in-person interactions, leading to substitutions away from offline shopping toward online shopping while maintaining the same level of overall spending on net. This could inflate the effect estimate. On the other hand, our credit card data do not include purchases from all payment processors so some of the shifted online purchases may not be captured in our data. To the extent that this is the case, it would bias our results toward zero.

**Closing Remarks**

We are grateful to the Editor and reviewers for the thorough and constructive review process. We believe the manuscript is substantially stronger as a result of these revisions. We look forward to hearing from the Editorial Office regarding our responses and revisions.
