# Supplementary material for: The impact of state- versus county-level mask mandates on economic activity during the COVID-19 pandemic
Source: PLoS One. 2026 Sep 3;21(9):e0332243. doi: 10.1371/journal.pone.0332243 (PMC13541194; doi:10.1371/journal.pone.0332243)
Supplement: Peer review history

# Peer review: Decision Letter 0

**DOI:** 10.1371/journal.pone.0332243.r001

**Authors:** Hongbum Kim

22 Dec 2025

Dear Dr. Nelson,

Thank you for submitting your manuscript to PLOS ONE. After careful consideration, we feel that it has merit but does not fully meet PLOS ONE’s publication criteria as it currently stands. Therefore, we invite you to submit a revised version of the manuscript that addresses the points raised during the review process.

Please submit your revised manuscript by Feb 05 2026 11:59PM. If you will need more time than this to complete your revisions, please reply to this message or contact the journal office at plosone@plos.org. When you're ready to submit your revision, log on to https://www.editorialmanager.com/pone/ and select the 'Submissions Needing Revision' folder to locate your manuscript file.

- A letter that responds to each point raised by the academic editor and reviewer(s). You should upload this letter as a separate file labeled 'Response to Reviewers'.
- A marked-up copy of your manuscript that highlights changes made to the original version. You should upload this as a separate file labeled 'Revised Manuscript with Track Changes'.
- An unmarked version of your revised paper without tracked changes. You should upload this as a separate file labeled 'Manuscript'.

If you would like to make changes to your financial disclosure, please include your updated statement in your cover letter. Guidelines for resubmitting your figure files are available below the reviewer comments at the end of this letter.

If applicable, we recommend that you deposit your laboratory protocols in protocols.io to enhance the reproducibility of your results. Protocols.io assigns your protocol its own identifier (DOI) so that it can be cited independently in the future. For instructions see: https://journals.plos.org/plosone/s/submission-guidelines#loc-laboratory-protocols. Additionally, PLOS ONE offers an option for publishing peer-reviewed Lab Protocol articles, which describe protocols hosted on protocols.io. Read more information on sharing protocols at https://plos.org/protocols?utm_medium=editorial-email&utm_source=authorletters&utm_campaign=protocols.

We look forward to receiving your revised manuscript.

Kind regards,

Hongbum Kim, Ph.D.

Academic Editor

PLOS One

Journal requirements:

When submitting your revision, we need you to address these additional requirements.

1. Please ensure that your manuscript meets PLOS ONE's style requirements, including those for file naming. The PLOS ONE style templates can be found at

https://journals.plos.org/plosone/s/file?id=wjVg/PLOSOne_formatting_sample_main_body.pdf and

https://journals.plos.org/plosone/s/file?id=ba62/PLOSOne_formatting_sample_title_authors_affiliations.pdf

2. Thank you for stating the following financial disclosure:

“Utah. This work was supported by the Centers for Disease Control and Prevention (grant number 1 NU38FT000009) and the State of Utah Governor’s Office of Management and Budget.”

Please state what role the funders took in the study.  If the funders had no role, please state: "The funders had no role in study design, data collection and analysis, decision to publish, or preparation of the manuscript."

If this statement is not correct you must amend it as needed.

Please include this amended Role of Funder statement in your cover letter; we will change the online submission form on your behalf.

3. Thank you for stating the following in the Acknowledgments Section of your manuscript:

“This material is the result of work supported with resources and the use of facilities at the George E. Wahlen Department of Veterans Affairs Medical Center, Salt Lake City, Utah. This work was supported by the Centers for Disease Control and Prevention (grant number 1 NU38FT000009) and the State of Utah Governor’s Office of Management and Budget. The funders had no role in study design, data collection and analysis, decision to publish, or preparation of the manuscript. The views expressed in this paper are those of the authors and do not necessarily represent the position or policy of the U.S. Department of Veterans Affairs or the United States Government.”

We note that you have provided funding information that is currently declared in your Funding Statement. However, funding information should not appear in the Acknowledgments section or other areas of your manuscript. We will only publish funding information present in the Funding Statement section of the online submission form.

Please remove any funding-related text from the manuscript and let us know how you would like to update your Funding Statement. Currently, your Funding Statement reads as follows:

“Utah. This work was supported by the Centers for Disease Control and Prevention (grant number 1 NU38FT000009) and the State of Utah Governor’s Office of Management and Budget.”

Please include your amended statements within your cover letter; we will change the online submission form on your behalf.

4. We note that you have indicated that there are restrictions to data sharing for this study. PLOS only allows data to be available upon request if there are legal or ethical restrictions on sharing data publicly. For more information on unacceptable data access restrictions, please see http://journals.plos.org/plosone/s/data-availability#loc-unacceptable-data-access-restrictions.

Before we proceed with your manuscript, please address the following prompts:

a) If there are ethical or legal restrictions on sharing a de-identified data set, please explain them in detail (e.g., data contain potentially identifying or sensitive patient information, data are owned by a third-party organization, etc.) and who has imposed them (e.g., a Research Ethics Committee or Institutional Review Board, etc.). Please also provide contact information for a data access committee, ethics committee, or other institutional body to which data requests may be sent.

b) If there are no restrictions, please upload the minimal anonymized data set necessary to replicate your study findings to a stable, public repository and provide us with the relevant URLs, DOIs, or accession numbers. For a list of recommended repositories, please see

https://journals.plos.org/plosone/s/recommended-repositories. You also have the option of uploading the data as Supporting Information files, but we would recommend depositing data directly to a data repository if possible.

We will update your Data Availability statement on your behalf to reflect the information you provide.

5. We note that Figure 1 in your submission contain [map/satellite] images which may be copyrighted. All PLOS content is published under the Creative Commons Attribution License (CC BY 4.0), which means that the manuscript, images, and Supporting Information files will be freely available online, and any third party is permitted to access, download, copy, distribute, and use these materials in any way, even commercially, with proper attribution. For these reasons, we cannot publish previously copyrighted maps or satellite images created using proprietary data, such as Google software (Google Maps, Street View, and Earth). For more information, see our copyright guidelines: http://journals.plos.org/plosone/s/licenses-and-copyright.

We require you to either (1) present written permission from the copyright holder to publish these figures specifically under the CC BY 4.0 license, or (2) remove the figures from your submission:

1. You may seek permission from the original copyright holder of Figure 1 to publish the content specifically under the CC BY 4.0 license.

We recommend that you contact the original copyright holder with the Content Permission Form (http://journals.plos.org/plosone/s/file?id=7c09/content-permission-form.pdf) and the following text:

“I request permission for the open-access journal PLOS ONE to publish XXX under the Creative Commons Attribution License (CCAL) CC BY 4.0 (http://creativecommons.org/licenses/by/4.0/). Please be aware that this license allows unrestricted use and distribution, even commercially, by third parties. Please reply and provide explicit written permission to publish XXX under a CC BY license and complete the attached form.”

Please upload the completed Content Permission Form or other proof of granted permissions as an "Other" file with your submission.

In the figure caption of the copyrighted figure, please include the following text: “Reprinted from [ref] under a CC BY license, with permission from [name of publisher], original copyright [original copyright year].”

2. If you are unable to obtain permission from the original copyright holder to publish these figures under the CC BY 4.0 license or if the copyright holder’s requirements are incompatible with the CC BY 4.0 license, please either i) remove the figure or ii) supply a replacement figure that complies with the CC BY 4.0 license. Please check copyright information on all replacement figures and update the figure caption with source information. If applicable, please specify in the figure caption text when a figure is similar but not identical to the original image and is therefore for illustrative purposes only.

The following resources for replacing copyrighted map figures may be helpful:

USGS National Map Viewer (public domain): http://viewer.nationalmap.gov/viewer/

The Gateway to Astronaut Photography of Earth (public domain): http://eol.jsc.nasa.gov/sseop/clickmap/

Maps at the CIA (public domain): https://www.cia.gov/library/publications/the-world-factbook/index.html and https://www.cia.gov/library/publications/cia-maps-publications/index.html

NASA Earth Observatory (public domain): http://earthobservatory.nasa.gov/

Landsat: http://landsat.visibleearth.nasa.gov/

USGS EROS (Earth Resources Observatory and Science (EROS) Center) (public domain): http://eros.usgs.gov/#

Natural Earth (public domain): http://www.naturalearthdata.com/

6. We notice that your supplementary figures are included in the manuscript file. Please remove them and upload them with the file type 'Supporting Information'. Please ensure that each Supporting Information file has a legend listed in the manuscript after the references list.

If the reviewer comments include a recommendation to cite specific previously published works, please review and evaluate these publications to determine whether they are relevant and should be cited. There is no requirement to cite these works unless the editor has indicated otherwise.

[Note: HTML markup is below. Please do not edit.]

Reviewers' comments:

Reviewer's Responses to Questions

**Comments to the Author**

1. Is the manuscript technically sound, and do the data support the conclusions?

Reviewer #1: Yes

Reviewer #2: Yes

Reviewer #3: Partly

**********

2. Has the statistical analysis been performed appropriately and rigorously?

Reviewer #1: Yes

Reviewer #2: Yes

Reviewer #3: Yes

**********

3. Have the authors made all data underlying the findings in their manuscript fully available?

The PLOS Data policy

Reviewer #1: Yes

Reviewer #2: Yes

Reviewer #3: Yes

**********

4. Is the manuscript presented in an intelligible fashion and written in standard English?

Reviewer #1: Yes

Reviewer #2: Yes

Reviewer #3: Yes

**********

Reviewer #1: The article is well aligned with its stated objective, and the findings hold significant relevance for policymakers seeking to design future responses to pandemics such as COVID-19. The comparative insights between state-level and nationwide interventions provide valuable guidance for evidence-based decision-making.

Reviewer #2: The paper shares an important question but the argument and analysis needs classification and restructuring of the theoretical analysis aligning with the results.

1) The paper hypothesize that county-level mandates provide a more potent, geographically specific signal about local virus risk. In the discussion, they posit that this increased perception of risk decreases economic activity. On the other hand, the empirical findings show that state-level mandates were associated with an increase in economic activity and spending, while county-level mandates had no statistically significant effect. Why does a weaker risk signal lead to a positive change in behavior, while a stronger signal leads to no change? Need further explanation for this.

2) Based on the inconsistency above in feedback 1), the interpretation should be rearranged. It could be that the county-level mandate's strong risk signal may be canceled out by its localized safety signal, resulting in an insignificant effect. This or an alternative analysis is needed to be integrated into the paper.

3) (Data) If credit card data does not distinguish between online and offline spending, could a shift from offline to online spending appear as positive (or insignificant) effect on 'spending' while representing a drop in the local economic activity (such as in restaurants)? If this cannot be clarified, this should be further mentioned in the limitation part.

Reviewer #3: 1. In the abstract, please use the full form instead of abbreviations (e.g., write the full term rather than using “vs.”).

2. On page 3, just before the discussion of signaling theory, provide a brief explanation of economic activity, which is the key variable of interest in this study.

3. On page 4, please include an appropriate reference for signaling theory.

4. On page 5, provide more detailed information about the data. For example, clarify whether the panel dataset is constructed at the county or state level, and explain the rationale for this choice.

5. In the Methods section, the econometric models should be explained more clearly. Specifically, provide a brief explanation of the regression discontinuity and difference-in-differences approaches, and justify why these methods are suitable for addressing the research question.

6. Please provide references to support the inclusion of elections as a confounding factor. Explain why elections are expected to influence the outcome variable. In addition, consider discussing other potentially important confounders that have not been addressed, such as employment status, income, COVID-19 exposure, age, gender, and other relevant socioeconomic or demographic factors.

7. If it is not a requirement of the journal to place figures and tables at the end of the manuscript, consider embedding them within the main text and updating the discussion accordingly.

8. In the Results and Discussion section, please interpret the estimated coefficients in substantive terms. For example, explain what values such as 1.05, −1.01, or 143.46 imply for the outcome variable and how they should be understood by readers.

**********

[NOTE: If reviewer comments were submitted as an attachment file, they will be attached to this email and accessible via the submission site. Please log into your account, locate the manuscript record, and check for the action link "View Attachments". If this link does not appear, there are no attachment files.]

To ensure your figures meet our technical requirements, please review our figure guidelines: https://journals.plos.org/plosone/s/figures

You may also use PLOS’s free figure tool, NAAS, to help you prepare publication quality figures: https://journals.plos.org/plosone/s/figures#loc-tools-for-figure-preparation.

NAAS will assess whether your figures meet our technical requirements by comparing each figure against our figure specifications.

# Author comment: Author response to Decision Letter 1

**DOI:** 10.1371/journal.pone.0332243.r002

2 Mar 2026

Part A. Editorial Letter and Journal Requirements

Comment:

Thank you for submitting your manuscript to PLOS ONE. After careful consideration, we feel that it has merit but does not fully meet PLOS ONE’s publication criteria as it currently stands. Therefore, we invite you to submit a revised version of the manuscript that addresses the points raised during the review process.

Response:

We appreciate the opportunity to revise. In the revised manuscript, we (i) reorganize and clarify the conceptual interpretation of our empirical results, (ii) expand and clarify the data description and unit of analysis, (iii) improve exposition of the econometric methods (regression discontinuity and difference-in-differences) and why they are appropriate, and (iv) add discussion and citations regarding confounding factors (including elections). We also implement the journal’s formatting and data/figure requirements described below.

Comment:

1. Please ensure that your manuscript meets PLOS ONE’s style requirements, including those for file naming.

Response:

We have updated the manuscript files to comply with PLOS ONE style and file-naming requirements and will upload: (i) a clean manuscript file, (ii) a marked-up manuscript with tracked changes, and (iii) this response-to-reviewers letter as a separate file.

Comment:

2. Thank you for stating the following financial disclosure: “Utah. This work was supported by the Centers for Disease Control and Prevention (grant number 1 NU38FT000009) and the State of Utah Governor’s Office of Management and Budget.” Please state what role the funders took in the study. If the funders had no role, please state: “The funders had no role in study design, data collection and analysis, decision to publish, or preparation of the manuscript.” Please include this amended Role of Funder statement in your cover letter; we will change the online submission form on your behalf.

Response:

We will include the requested Role of Funder statement in the cover letter submitted through Editorial Manager. Specifically, we will state: “The funders had no role in study design, data collection and analysis, decision to publish, or preparation of the manuscript.” If any aspect of that statement requires modification based on internal documentation, we will amend accordingly in the cover letter so the submission form can be updated.

Comment:

3. We note that you have provided funding information that is currently declared in your Funding Statement. However, funding information should not appear in the Acknowledgments section or other areas of your manuscript. Please remove any funding-related text from the manuscript and let us know how you would like to update your Funding Statement. Currently, your Funding Statement reads as follows: “Utah. This work was supported by the Centers for Disease Control and Prevention (grant number 1 NU38FT000009) and the State of Utah Governor’s Office of Management and Budget.”

Response:

We have removed funding-related text from the Acknowledgments section (and any other manuscript locations) so that funding information appears only in the Funding Statement section of the online submission form. In the cover letter, we will (i) confirm the preferred Funding Statement text for publication and (ii) request any necessary updates to the online Funding Statement to match that preferred wording.

Comment:

4. We note that you have indicated that there are restrictions to data sharing for this study. PLOS only allows data to be available upon request if there are legal or ethical restrictions on sharing data publicly. (a) If there are ethical or legal restrictions, please explain them and provide contact information for the relevant data access committee/IRB/etc. (b) If there are no restrictions, please deposit the minimal anonymized data set necessary to replicate your findings in a stable public repository and provide URLs/DOIs/accession numbers.

Response:

We will revise the Data Availability statement to comply with PLOS ONE policy and will provide the requested information. Specifically, we will describe the legal/ethical restrictions in detail and provide contact information for the institutional body responsible for data access requests; and we will provide synthetic data upon request. We have included the final language in the cover letter so that PLOS ONE can update the Data Availability statement in the submission system.

Comment:

5. We note that Figure 1 contains [map/satellite] images which may be copyrighted. PLOS cannot publish copyrighted maps or satellite images created using proprietary data, such as Google software. We require you to either present written permission to publish these figures under CC BY 4.0, or remove/replace the figures with compliant alternatives.

Response:

Figure 1 is not a copyrighted map or satellite image. It is produced using Stata based on the authors’ data.

Comment:

6. We notice that your supplementary figures are included in the manuscript file. Please remove them and upload them with the file type ’Supporting Information’. Please ensure that each Supporting Information file has a legend listed in the manuscript after the references list.

Response:

We have removed supplementary figures from the main manuscript file. We will upload them as separate Supporting Information files with the correct file type designation, and we have added a Supporting Information legend section.

Part B. Reviewer Comments

Reviewer #1

Comment:

The article is well aligned with its stated objective, and the findings hold significant relevance for policymakers seeking to design future responses to pandemics such as COVID-19. The comparative insights between state-level and nationwide interventions provide valuable guidance for evidence-based decision-making.

Response:

We appreciate this supportive assessment. In the revised manuscript, we preserve the focus on policy relevance while strengthening the clarity and structure of the conceptual framing and interpretation, and we add additional details on data construction and empirical identification to improve transparency and reproducibility.

Reviewer #2

Comment:

1) The paper hypothesize that county-level mandates provide a more potent, geographically specific signal about local virus risk. In the discussion, they posit that this increased perception of risk decreases economic activity. On the other hand, the empirical findings show that state-level mandates were associated with an increase in economic activity and spending, while county-level mandates had no statistically significant effect. Why does a weaker risk signal lead to a positive change in behavior, while a stronger signal leads to no change? Need further explanation for this.

Response:

Thank you for giving us the opportunity to clarify. We focus on the signal effects, as you highlight. There is another effect. In particular, mask mandates increase mask wearing in public and make activity safer. We find that this direct effect is larger than the signal effect for state mandates because the signal is weaker with state mandates. In contrast, the direct effect is smaller than the signal effect for county mandates, leading to a decrease in activity and spending. We now explain this in the paper when discussing Figure 2. Specifically, we state, “The mask mandates have two effects: they increase safety by increasing mask wearing in public, encouraging activity and spending, and they signal increased risk, discouraging activity and spending. On net, we find the signal is sufficiently weak with state mandates to lead to an increase in activity and spending, while the signal of risk is sufficiently strong with county mandates such that the information effect is larger

than the effect of increased safety.”

Comment:

2) Based on the inconsistency above in feedback 1), the interpretation should be rearranged. It could be that the county-level mandate’s strong risk signal may be canceled out by its localized safety signal, resulting in an insignificant effect. This or an alternative analysis is needed to be integrated into the paper.

Response:

We agree that the net effect of the risk signal and increased safety is ambiguous for both county and state mandates, which motivates our empirical analysis. Our preferred interpretation is that i) the risk signal is bigger for county mandates and state mandates and ii) the safety effect is similar for county and state mandates (they have similar increases in mask wearing). If this is the case, then we should see relatively more activity with state mandates than county mandates—but this could have manifested in an overall decrease in activity for both state and county mandates, an overall increase in activity for both state and county mandates, or, as we find, an increase for state mandates and a decrease for county mandates. Said differently, theory suggests the relative strength of the risk signal but the net effect is ambiguous and must be estimated empirically.

Comment:

3) (Data) If credit card data does not distinguish between online and offline spending, could a shift from offline to online spending appear as positive (or insignificant) effect on ’spending’ while representing a drop in the local economic activity (such as in restaurants)? If this cannot be clarified, this should be further mentioned in the limitation part.

Response:

We now note the limitation of the data that we cannot distinguish online and offline spending. We suggest this limitation adds noise the spending estimates and is one reason for the sharper contrast in the activity estimates. We now state, “It is important to point out several limitations to our study. First, credit card spending and cell phone data – while useful measures of purchasing and mobility, respectively – do not capture the entirety of economic activity. In addition, the credit card spending does not distinguish between online and offline spending.”

Reviewer #3

Comment:

1. In the abstract, please use the full form instead of abbreviations (e.g., write the full term rather than using “vs.”).

Response:

Done. We revised the abstract to eliminate informal abbreviations (e.g., “vs.”) and to use full terms throughout.

Comment:

2. On page 3, just before the discussion of signaling theory, provide a brief explanation of economic activity, which is the key variable of interest in this study.

Response:

Done. We added a brief definition and explanation of our economic activity outcome measures immediately prior to introducing signaling theory. Specifically, we state, “As motivation for examining this relationship, it is helpful to consider the mechanism by which mask mandates might influence economic activity, which we measure as cell phone mobility and credit card spending.” Thank you for this suggestion, we hope it clarifies for readers upfront our measure and leaves more details for later.

Comment:

3. On page 4, please include an appropriate reference for signaling theory.

Response:

Done. We added citations to foundational and widely used references in the signaling literature Stiglitz 2002 in the American Economic Review (2,979 citations).

Comment:

4. On page 5, provide more detailed information about the data. For example, clarify whether the panel dataset is constructed at the county or state level, and explain the rationale for this choice.

Response:

Done. Specifically, we state, “We construct the dataset at the county level because that is the finest level we can obtain for many sources.”

Comment:

5. In the Methods section, the econometric models should be explained more clearly. Specifically, provide a brief explanation of the regression discontinuity and difference-in-differences approaches, and justify why these methods are suitable for addressing the research question.

Response:

Done. We now include two paragraphs at the beginning of the statistical analyses section that briefly explain the regression discontinuity and difference-in-differences approaches. We also justify why these methods are suitable for addressing our research question. Specifically, we now state, “We employ multiple empirical strategies, including a regression discontinuity (RD) design and a difference-in-differences (DiD) approach. The RD design exploits the discrete timing of mask mandate implementation by comparing activity in counties immediately before and after the policy change, under the assumption that unobserved determinants of activity evolve smoothly at the cutoff. This allows us to isolate the local effect of mandate adoption. We then contrast these discontinuous changes between counties subject to county-level mandates and those subject to state-level mandates.

The DiD approach complements the RD analysis by comparing trends in activity between counties with state and county mask mandates over a longer horizon. Prior to mandate adoption, counties that ultimately receive state mandates exhibit similar activity trends to those with county mandates, supporting the parallel trends assumption. The DiD estimates capture differential changes in activity following mandate implementation, while staggered adoption across counties provides additional identifying variation and mitigates concerns about confounding time-varying shocks. Together, these methods allow us to assess whether county and state mandates differ in their signaling effects on economic activity.”

Comment:

6. Please provide references to support the inclusion of elections as a confounding factor. Explain why elections are expected to influence the outcome variable. In addition, consider discussing other potentially important confounders that have not been addressed, such as employment status, income, COVID-19 exposure, age, gender, and other relevant socioeconomic or demographic factors.

Response:

We added citations and explanation for the inclusion of elections as a potential confounder (e.g., elections can shift mobility, spending, public gatherings, messaging, and policy behavior). We also expanded the confounders discussion to acknowledge and, where feasible, incorporate additional relevant factors (e.g., labor market conditions, income, demographic composition, and COVID-19 exposure). Where such factors cannot be directly observed in our data, we discuss the potential direction of bias and clarify limitations.

Comment:

7. If it is not a requirement of the journal to place figures and tables at the end of the manuscript, consider embedding them within the main text and updating the discussion accordingly.

Response:

We have updated the presentation of figures and tables in alignment with PLOS ONE conventions. Supplementary figures are now provided as Supporting Information files, per journal guidance.

Comment:

8. In the Results and Discussion section, please interpret the estimated coefficients in substantive terms. For example, explain what values such as 1.05, −1.01, or 143.46 imply for the outcome variable and how they should be understood by readers.

Response:

We thank the referee for this suggestion. We have revised the Results and Discussion sections to provide substantive interpretations of key coefficients. Specifically, we now explain how estimates such as 1.05, 1.01, and 143.46 translate into percentage-point changes in mobility and dollar changes in monthly spending, and we benchmark these magnitudes relative to sample means to aid interpretation.

Specifically, we now state, “The estimated RD coefficient of 1.05 for activity implies that, following a state mask mandate, county-level mobility increased by approximately 1.05 percentage points relative to the same calendar day in 2019. Given that average activity during our sample period was roughly 14–16 percent below 2019 levels, this effect corresponds to a non-trivial rebound in economic activity. In contrast, the estimated coefficient of 1.01 following county mask mandates indicates a small decline in activity of approximately one percentage point, though this estimate is not statistically distinguishable from zero. This suggests that county mandates did not generate meaningful changes in observed mobility around the time of implementation. For spending outcomes, the estimated coefficient

# Peer review: Decision Letter 1

**DOI:** 10.1371/journal.pone.0332243.r003

**Authors:** Hongbum Kim

8 Apr 2026

Dear Dr. Nelson,

Thank you for submitting your manuscript to PLOS ONE. After careful consideration, we feel that it has merit but does not fully meet PLOS ONE’s publication criteria as it currently stands. Therefore, we invite you to submit a revised version of the manuscript that addresses the points raised during the review process.

Please submit your revised manuscript by May 23 2026 11:59PM. If you will need more time than this to complete your revisions, please reply to this message or contact the journal office at plosone@plos.org. When you're ready to submit your revision, log on to https://www.editorialmanager.com/pone/ and select the 'Submissions Needing Revision' folder to locate your manuscript file.

- A letter that responds to each point raised by the academic editor and reviewer(s). You should upload this letter as a separate file labeled 'Response to Reviewers'.
- A marked-up copy of your manuscript that highlights changes made to the original version. You should upload this as a separate file labeled 'Revised Manuscript with Track Changes'.
- An unmarked version of your revised paper without tracked changes. You should upload this as a separate file labeled 'Manuscript'.

If you would like to make changes to your financial disclosure, please include your updated statement in your cover letter. Guidelines for resubmitting your figure files are available below the reviewer comments at the end of this letter.

If applicable, we recommend that you deposit your laboratory protocols in protocols.io to enhance the reproducibility of your results. Protocols.io assigns your protocol its own identifier (DOI) so that it can be cited independently in the future. For instructions see: https://journals.plos.org/plosone/s/submission-guidelines#loc-laboratory-protocols. Additionally, PLOS ONE offers an option for publishing peer-reviewed Lab Protocol articles, which describe protocols hosted on protocols.io. Read more information on sharing protocols at https://plos.org/protocols?utm_medium=editorial-email&utm_source=authorletters&utm_campaign=protocols.

As the corresponding author, your ORCID iD is verified in the submission system and will appear in the published article. PLOS supports the use of ORCID, and we encourage all coauthors to register for an ORCID iD and use it as well. Please encourage your coauthors to verify their ORCID iD within the submission system before final acceptance, as unverified ORCID iDs will not appear in the published article. *Only*  the individual author can complete the verification step; PLOS staff *cannot*  verify ORCID iDs on behalf of authors.

We look forward to receiving your revised manuscript.

Kind regards,

Hongbum Kim, Ph.D.

Academic Editor

PLOS One

Journal Requirements:

If the reviewer comments include a recommendation to cite specific previously published works, please review and evaluate these publications to determine whether they are relevant and should be cited. There is no requirement to cite these works unless the editor has indicated otherwise.

Additional Editor Comments (if provided):

Despite the positive evaluation of the revised manuscript by Reviewer #3, it appears that several issues have not yet been adequately addressed.

3-3. Do you believe that the inclusion of only one reference regarding the mention of signaling theory is sufficient?

3-5. The current explanation of econometric models is highly insufficient, mentioning these models without elaboration. You should add existing literature demonstrating the advantages of the methodology and specific equation models employed, so that readers can fully understand the manuscript.

3-6. The rationale and related interpretation regarding the election remain inadequate. A more detailed explanation is necessary to explain the rationale that this variable is included. Moreover, while readers in U.S. may readily understand the distinctions between the Democratic and Republican parties, this may not be intuitively grasped by researchers worldwide. An explanation addressing this issue should be included.

3-7. Why were “Supplementary Figures and Tables” organized? Since they contain critical interpretations to the paper as a whole, classifying them as supplementary materials implies that they are not important beyond the RD analysis. Please Include them as regular figures and tables, and as Reviewer 3 mentioned, place them within the main text rather than at the end of the text.

Additionally, the following points should be addressed.

1. The paper lacks references overall. In particular, Introduction and Discussion sections require the inclusion of more references supporting each argument to ensure the problem descriptions and analysis results. For example, literature regarding policy interventions, the impact of COVID-19, etc.

2. A mix of different font styles is found throughout the manuscript. Incomplete sentences as well. Please pay close attention to formatting as well.

3. In Supplementary Table 1, the R-squared value for Panel A (4) is 0.001, whereas for Panel B it is 0.883. Also, the R-squared value for Panel A (6) is 0.873, while for Panel B it is 0.002. Since the R-squared values for the other models are at similar levels, it is therefore necessary to check whether (4) and (6) can differ so drastically.

4. Discussion -> Discussion and Conclusion

5. It is noteworthy that all the references cited on page 16 were published in 2020. A coincidence or Intentional? It gives an awkward matter. Why were these papers not cited in the Introduction?

The Editorial Office plans to comprehensively evaluate whether the above points and Reviewer 2’s comment have been addressed, and proceed to make a final decision in the next round.

[Note: HTML markup is below. Please do not edit.]

Reviewers' comments:

Reviewer's Responses to Questions

**Comments to the Author**

Reviewer #2: All comments have been addressed

Reviewer #3: All comments have been addressed

**********

2. Is the manuscript technically sound, and do the data support the conclusions?

Reviewer #2: Yes

Reviewer #3: Yes

**********

3. Has the statistical analysis been performed appropriately and rigorously?

Reviewer #2: Yes

Reviewer #3: Yes

**********

4. Have the authors made all data underlying the findings in their manuscript fully available?

The PLOS Data policy

Reviewer #2: Yes

Reviewer #3: Yes

**********

5. Is the manuscript presented in an intelligible fashion and written in standard English?

Reviewer #2: Yes

Reviewer #3: Yes

**********

Reviewer #2: The authors added a limitation regarding not being able to distinguish between online and offline spending. Briefly discuss how this might bias the spending per person - results. (ex. potential substitution bias)

Reviewer #3: The comments have been addressed adequately - the manuscript has been improved sufficiently. I don't have any further comment.

**********

what does this mean?). If published, this will include your full peer review and any attached files.

If you choose “no”, your identity will remain anonymous but your review may still be made public.

**Do you want your identity to be public for this peer review?**  For information about this choice, including consent withdrawal, please see our Privacy Policy

Reviewer #2: No

Reviewer #3: **Yes**

**********

[NOTE: If reviewer comments were submitted as an attachment file, they will be attached to this email and accessible via the submission site. Please log into your account, locate the manuscript record, and check for the action link "View Attachments". If this link does not appear, there are no attachment files.]

To ensure your figures meet our technical requirements, please review our figure guidelines: https://journals.plos.org/plosone/s/figures

You may also use PLOS’s free figure tool, NAAS, to help you prepare publication quality figures: https://journals.plos.org/plosone/s/figures#loc-tools-for-figure-preparation.

NAAS will assess whether your figures meet our technical requirements by comparing each figure against our figure specifications.

# Author comment: Author response to Decision Letter 2

**DOI:** 10.1371/journal.pone.0332243.r004

19 May 2026

Responses to Additional Editor Comments

Editor Comment 3-3: Do you believe that the inclusion of only one reference regarding the mention of signaling theory is sufficient?

Response:

We agree that our earlier treatment of signaling theory was insufficiently grounded in the literature. We have expanded the discussion and now cite several foundational and applied works on signaling theory that are directly relevant to our context. Specifically, we have added citations for the following articles:

1. Akerlof, George A. "The Market for 'Lemons': Quality Uncertainty and the Market Mechanism." Quarterly Journal of Economics, 1970, 84(3), 488–500.

2. Cho, In-Koo and David M. Kreps. "Signaling Games and Stable Equilibria." Quarterly Journal of Economics, 1987, 102(2), 179–221.

3. Dranove, David and Ginger Zhe Jin. "Quality Disclosure and Certification: Theory and Practice." Journal of Economic Literature, 2010, 48(4), 935–963.

4. Grossman, Sanford J. and Joseph E. Stiglitz. "On the Impossibility of Informationally Efficient Markets." American Economic Review, 1980, 70(3), 393–408.

5. Jin, Ginger Zhe and Phillip Leslie. "The Effect of Information on Product Quality: Evidence from Restaurant Hygiene Grade Cards." Quarterly Journal of Economics, 2003, 118(2), 409–451.

6. Milgrom, Paul R. "Good News and Bad News: Representation Theorems and Applications." Bell Journal of Economics, 1981, 12(2), 380–391.

7. Morris, Stephen and Hyun Song Shin. "Social Value of Public Information." American Economic Review, 2002, 92(5), 1521–1534.

8. Spence, Michael. "Job Market Signaling." Quarterly Journal of Economics, 1973, 87(3), 355–374.

We relate our work to the literature through this paragraph we have added to the introduction:

“The theoretical framework of this paper draws on the economics of signaling and information disclosure. Akerlof (1970) and Spence (1973) establish the foundational insight that observable actions by informed parties transmit private information to less-informed parties and alter their behavior accordingly. Grossman and Stiglitz (1980) extend this logic to show that agents rationally expend resources to act on information precisely because policies and prices are imperfect aggregators of what the informed party knows. In our setting, government mask mandates function as credible signals: policymakers, who have access to localized disease surveillance data, reveal their private information about transmission risk through the act of mandate adoption. Cho and Kreps (1987) provide the equilibrium foundation for this interpretation—agents correctly infer that a mandate would only be enacted when conditions warranted it, since a policymaker with no cause for concern would not bear the political cost of a mandate. A key insight from Morris and Shin (2002) is then directly applicable: the precision of a public signal determines how strongly agents update their beliefs and coordinate behavior. A county-level mandate is a more precise signal than a state-level mandate because it draws on geographically finer information, leading agents to update beliefs about local transmission risk more sharply. Milgrom (1981) reinforces this logic through the unraveling result: when policymakers have private information and disclosure is credible, constituents infer the worst from inaction, making the decision to mandate highly informative. This precision, however, cuts both ways—a stronger signal of risk leads agents to curtail economic activity, partially offsetting the direct safety benefit of the mandate. The empirical literature on mandatory disclosure supports this behavioral response: Jin and Leslie (2003) show that county-mandated restaurant hygiene grade cards caused consumers to reallocate spending in response to revealed health risk, and Dranove and Jin (2010) document more broadly that government-mandated disclosure of health and quality information generates substantial and often unintended behavioral consequences. The divergence we document between state and county mandates is a direct implication of signal precision—more geographically targeted policies are more informative, produce larger belief updating, and in the case of mask mandates, generate a risk-avoidance response strong enough to reduce mobility and spending relative to the less precise state-level signal.”

We believe these additions provide a more thorough foundation for readers unfamiliar with this strand of theory.

Editor Comment 3-5: The current explanation of econometric models is highly insufficient, mentioning these models without elaboration. You should add existing literature demonstrating the advantages of the methodology and specific equation models employed, so that readers can fully understand the manuscript.

Response:

We thank the Editor for this important observation. We have substantially expanded the econometric methodology section. We now include: (1) a full exposition of the estimating equations, presented in numbered equation form; (2) a discussion of identification assumptions and why they are satisfied in our setting; and (3) citations to methodological literature that demonstrate the advantages of our chosen approach.

Editor Comment 3-6: The rationale and related interpretation regarding the election remain inadequate. A more detailed explanation is necessary. Moreover, while readers in the U.S. may readily understand the distinctions between the Democratic and Republican parties, this may not be intuitively grasped by researchers worldwide.

Response:

We appreciate this comment and recognize that our earlier treatment assumed too much background knowledge on the part of international readers. We now add this explanation in our independent variable section,

Political affiliation is an important confounder in this setting for several reasons. First, Allcott et al. (2020) document large and persistent partisan gaps in voluntary social distancing behavior during the COVID-19 pandemic, with residents of Democratic-leaning counties substantially more likely to reduce mobility and adopt protective behaviors independent of any formal mandate—meaning that baseline economic activity may differ systematically by political composition before a mandate ever takes effect. Second, because voluntary behavioral responses of this kind can reduce the measured discontinuity at the mandate threshold, failing to control for political affiliation would attenuate estimates in Democratic counties and inflate them in Republican counties, introducing differential bias across the two groups we compare. Third, political leaning correlates directly with the level of government enacting the mandate: in our sample, 45% of county-mandate counties voted Democratic compared to only 18% of state-mandate counties, meaning that partisan composition is a confounder not only of the mandate–activity relationship but specifically of the state-versus-county comparison that is central to our identification strategy.

Editor Comment 3-7: Why were “Supplementary Figures and Tables” organized as supplementary? Since they contain critical interpretations to the paper as a whole, classifying them as supplementary materials implies that they are not important beyond the RD analysis. Please include them as regular figures and tables within the main text.

Response:

We have now included the Supplementary Figures and Tables in the main body of the paper.

Responses to Additional Editorial Points

Editor Point 1: The paper lacks references overall. In particular, Introduction and Discussion sections require more references supporting each argument.

Response: Thank you. We have added more references to the Introduction, Methods, and Discussion sections.

Editor Point 2: A mix of different font styles is found throughout the manuscript. Incomplete sentences as well. Please pay close attention to formatting.

Response:

Thank you for bringing this to our attention. We have carefully read through the manuscript and ensured that all font styles are consistent and that sentences are complete.

Editor Point 3: In Supplementary Table 1, the R-squared value for Panel A (4) is 0.001, whereas for Panel B it is 0.883. Also, the R-squared for Panel A (6) is 0.873, while for Panel B it is 0.002. Please check whether these discrepancies are valid.

Response:

Thank you for flagging this. The discrepancies in R-squared values across panels are correct as reported. The variation reflects differences in fixed effect structure across specifications. In Panel A (4) and Panel B (6), the low R-squared values arise because those specifications include no fixed effects, so the regressors of interest explain little of the overall variance in the outcome. which is expected given the substantial residual heterogeneity across observations. In Panel A (6) and Panel B (4), by contrast, the high R-squared values are driven by rich fixed effect structures that absorb a large share of the variation in the dependent variable. This pattern, in which R-squared rises sharply with fixed-effect saturation, is a standard feature of panel regressions and does not indicate any inconsistency in the underlying estimates. We are happy to add a note to the table clarifying this if it would be helpful.

Editor Point 4: Discussion → Discussion and Conclusion.

Response:

Thank you for this suggestion. We have now added a Conclusion heading that comes before the final 2 paragraphs.

Editor Point 5: It is noteworthy that all references cited on page 16 were published in 2020. A coincidence or intentional? Why were these papers not cited in the Introduction?

Response:

The concentration of 2020 citations on page 16 was not intentional; these papers were cited at the point in the analysis where they were most directly relevant. However, we recognize that citing them only in the Discussion creates an unbalanced presentation. We have reviewed the Introduction and identified the appropriate places to cite these works earlier in the manuscript, noting their relevance to the problem description and motivating evidence.

Closing Remarks

We are grateful to the Editor and reviewers for the thorough and constructive review process. We believe the manuscript is substantially stronger as a result of these revisions. We look forward to hearing from the Editorial Office.

# Editor report: Decision Letter 2

**DOI:** 10.1371/journal.pone.0332243.r005

**Authors:** Hongbum Kim

1 Jun 2026

Dear Dr. Nelson,

Thank you for submitting your manuscript to PLOS ONE. After careful consideration, we feel that it has merit but does not fully meet PLOS ONE’s publication criteria as it currently stands. Therefore, we invite you to submit a revised version of the manuscript that addresses the points raised during the review process.

Please submit your revised manuscript by Jul 16 2026 11:59PM. If you will need more time than this to complete your revisions, please reply to this message or contact the journal office at plosone@plos.org. When you're ready to submit your revision, log on to https://www.editorialmanager.com/pone/ and select the 'Submissions Needing Revision' folder to locate your manuscript file.

- A letter that responds to each point raised by the academic editor and reviewer(s). You should upload this letter as a separate file labeled 'Response to Reviewers'.
- A marked-up copy of your manuscript that highlights changes made to the original version. You should upload this as a separate file labeled 'Revised Manuscript with Track Changes'.
- An unmarked version of your revised paper without tracked changes. You should upload this as a separate file labeled 'Manuscript'.

If you would like to make changes to your financial disclosure, please include your updated statement in your cover letter. Guidelines for resubmitting your figure files are available below the reviewer comments at the end of this letter.

If applicable, we recommend that you deposit your laboratory protocols in protocols.io to enhance the reproducibility of your results. Protocols.io assigns your protocol its own identifier (DOI) so that it can be cited independently in the future. For instructions see: https://journals.plos.org/plosone/s/submission-guidelines#loc-laboratory-protocols. Additionally, PLOS ONE offers an option for publishing peer-reviewed Lab Protocol articles, which describe protocols hosted on protocols.io. Read more information on sharing protocols at https://plos.org/protocols?utm_medium=editorial-email&utm_source=authorletters&utm_campaign=protocols.

As the corresponding author, your ORCID iD is verified in the submission system and will appear in the published article. PLOS supports the use of ORCID, and we encourage all coauthors to register for an ORCID iD and use it as well. Please encourage your coauthors to verify their ORCID iD within the submission system before final acceptance, as unverified ORCID iDs will not appear in the published article. *Only*  the individual author can complete the verification step; PLOS staff *cannot*  verify ORCID iDs on behalf of authors.

We look forward to receiving your revised manuscript.

Kind regards,

Hongbum Kim, Ph.D.

Academic Editor

PLOS One

Journal Requirements:

If the reviewer comments include a recommendation to cite specific previously published works, please review and evaluate these publications to determine whether they are relevant and should be cited. There is no requirement to cite these works unless the editor has indicated otherwise.

Please review your reference list to ensure that it is complete and correct. If you have cited papers that have been retracted, please include the rationale for doing so in the manuscript text, or remove these references and replace them with relevant current references. Any changes to the reference list should be mentioned in the rebuttal letter that accompanies your revised manuscript. If you need to cite a retracted article, indicate the article’s retracted status in the References list and also include a citation and full reference for the retraction notice.

Additional Editor Comments:

Response to Reviewer #2's comment is not provided. Please address Reviewer #2's concern.

I did not mean to split Discussion and Conclusion independently. Please revise the last section title as "Discussion and Conclusion."

[Note: HTML markup is below. Please do not edit.]

[NOTE: If reviewer comments were submitted as an attachment file, they will be attached to this email and accessible via the submission site. Please log into your account, locate the manuscript record, and check for the action link "View Attachments". If this link does not appear, there are no attachment files.]

To ensure your figures meet our technical requirements, please review our figure guidelines: https://journals.plos.org/plosone/s/figures

You may also use PLOS’s free figure tool, NAAS, to help you prepare publication quality figures: https://journals.plos.org/plosone/s/figures#loc-tools-for-figure-preparation.

NAAS will assess whether your figures meet our technical requirements by comparing each figure against our figure specifications.

# Author comment: Author response to Decision Letter 3

**DOI:** 10.1371/journal.pone.0332243.r006

2 Jul 2026

Responses to Editor Comments

Editor Comment: Response to Reviewer #2’s comment is not provided. Please address Reviewer #2’s concern.

Response:

Thank you for catching this and apologies for missing this on our previous submission. Please find our response below.

Editor Comment: I did not mean to split Discussion and Conclusion independently. Please revise the last section title as “Discussion and Conclusion”

Response:

We have made this change in the revised version of the manuscript.

Responses to Reviewer #2 Comment

Comment: The authors added a limitation regarding not being able to distinguish between online and offline spending. Briefly discuss how this might bias the spending per person - results. (ex. potential substitution bias).

Response: We appreciate the reviewer asking for more detail on this limitation. We have expanded the description of the consequences of our inability to distinguish between online and offline spending in the discussion section. To briefly summarize, the direction of the possible substitution bias could go either way. Mask mandates may increase the perceived risk of in-person interactions, leading to substitutions away from offline shopping toward online shopping while maintaining the same level of overall spending on net. This could inflate the effect estimate. On the other hand, our credit card data do not include purchases from all payment processors so some of the shifted online purchases may not be captured in our data. To the extent that this is the case, it would bias our results toward zero.

Closing Remarks

We are grateful to the Editor and reviewers for the thorough and constructive review process. We believe the manuscript is substantially stronger as a result of these revisions. We look forward to hearing from the Editorial Office regarding our responses and revisions.

Attachment Submitted filename: Response to Editor.docx

# Peer review: Decision Letter 3

**DOI:** 10.1371/journal.pone.0332243.r007

**Authors:** Hongbum Kim

16 Jul 2026

The Impact of State- versus County-Level Mask Mandates on Economic Activity During the COVID-19 Pandemic

PONE-D-25-46516R3

Dear Dr. Nelson,

We’re pleased to inform you that your manuscript has been judged scientifically suitable for publication and will be formally accepted for publication once it meets all outstanding technical requirements.

Within one week, you’ll receive an e-mail detailing the required amendments. When these have been addressed, you’ll receive a formal acceptance letter and your manuscript will be scheduled for publication.

An invoice will be generated when your article is formally accepted. Please note, if your institution has a publishing partnership with PLOS and your article meets the relevant criteria, all or part of your publication costs will be covered. Please make sure your user information is up-to-date by logging into Editorial Manager at Editorial Manager® and clicking the ‘Update My Information' link at the top of the page. For questions related to billing, please contact billing support.

If your institution or institutions have a press office, please notify them about your upcoming paper to help maximize its impact. If they’ll be preparing press materials, please inform our press team as soon as possible -- no later than 48 hours after receiving the formal acceptance. Your manuscript will remain under strict press embargo until 2 pm Eastern Time on the date of publication. For more information, please contact onepress@plos.org.

Kind regards,

Hongbum Kim, Ph.D.

Academic Editor

PLOS One

Additional Editor Comments (optional):

Thank you for your efforts on revising the manuscript. All comments are relevantly addressed, except some formatting issues. I hope these issues will be resolved in proofreading step.

Reviewers' comments:

Reviewer's Responses to Questions

**Comments to the Author**

Reviewer #2: All comments have been addressed

**********

2. Is the manuscript technically sound, and do the data support the conclusions?

Reviewer #2: Yes

**********

3. Has the statistical analysis been performed appropriately and rigorously?

Reviewer #2: Yes

**********

4. Have the authors made all data underlying the findings in their manuscript fully available?

The PLOS Data policy

Reviewer #2: Yes

**********

5. Is the manuscript presented in an intelligible fashion and written in standard English?

Reviewer #2: Yes

**********

Reviewer #2: All comments have been addressed including the one for Review 2. The author(s) have done a consistent job in making the article comprehensive, while addressing the underlying limitations. Well done.

**********

what does this mean?). If published, this will include your full peer review and any attached files.

If you choose “no”, your identity will remain anonymous but your review may still be made public.

**Do you want your identity to be public for this peer review?**  For information about this choice, including consent withdrawal, please see our Privacy Policy

Reviewer #2: **Yes**

**********

# Editor report: Acceptance letter

**DOI:** 10.1371/journal.pone.0332243.r008

**Authors:** Hongbum Kim

PONE-D-25-46516R3

PLOS One

Dear Dr. Nelson,

I'm pleased to inform you that your manuscript has been deemed suitable for publication in PLOS One. Congratulations! Your manuscript is now being handed over to our production team.

At this stage, our production department will prepare your paper for publication. This includes ensuring the following:

* All references, tables, and figures are properly cited

* All relevant supporting information is included in the manuscript submission,

* There are no issues that prevent the paper from being properly typeset

You will receive further instructions from the production team, including instructions on how to review your proof when it is ready. Please keep in mind that we are working through a large volume of accepted articles, so please give us a few days to review your paper and let you know the next and final steps.

Lastly, if your institution or institutions have a press office, please let them know about your upcoming paper now to help maximize its impact. If they'll be preparing press materials, please inform our press team within the next 48 hours. Your manuscript will remain under strict press embargo until 2 pm Eastern Time on the date of publication. For more information, please contact onepress@plos.org.

You will receive an invoice from PLOS for your publication fee after your manuscript has reached the completed accept phase. If you receive an email requesting payment before acceptance or for any other service, this may be a phishing scheme. Learn how to identify phishing emails and protect your accounts at https://explore.plos.org/phishing.

If we can help with anything else, please email us at customercare@plos.org.

Thank you for submitting your work to PLOS ONE and supporting open access.

Kind regards,

PLOS ONE Editorial Office Staff

on behalf of

Professor Hongbum Kim

Academic Editor

PLOS One
